# Microstructural Contributions of Different Polyolefins to the Deformation Mechanisms of Their Binary Blends

**DOI:** 10.3390/polym12051171

**Published:** 2020-05-20

**Authors:** Astrid Van Belle, Ruben Demets, Nicolas Mys, Karen Van Kets, Jo Dewulf, Kevin Van Geem, Steven De Meester, Kim Ragaert

**Affiliations:** 1 Centre for Polymer and Material Technologies (CPMT), Department of Materials, Textiles and Chemical Engineering, Faculty of Engineering and Architecture, Ghent University, Technologiepark 130, B-9052 Zwijnaarde, Belgium; van.belle.astrid@gmail.com (A.V.B.); ruben.demets@ugent.be (R.D.); nicolas.mys@ugent.be (N.M.); karen.vankets@ugent.be (K.V.K.); 2 Department of Green Chemistry and Technology, Faculty of Bioscience Engineering, Ghent University—Campus Kortrijk, Graaf Karel de Goedelaan 5, 8500 Kortrijk, Belgium; steven.demeester@ugent.be; 3 Sustainable Systems Engineering (STEN), Department of Green Chemistry and Technology, Faculty of Bioscience Engineering, Ghent University, Coupure Links 653, 9000 Ghent, Belgium; jo.dewulf@ugent.be; 4 Laboratory for Chemical Technology (LCT), Department of Materials, Textiles and Chemical Engineering, Faculty of Engineering and Architecture, Ghent University, Technologiepark 125, B-9052 Zwijnaarde, Belgium; kevin.vangeem@ugent.be

**Keywords:** immiscible polymer blends, polyolefins, deformation mechanisms, commodity plastics, mechanical recycling, structure–property relationships

## Abstract

The mixing of polymers, even structurally similar polyolefins, inevitably leads to blend systems with a phase-separated morphology. Fundamentally understanding the changes in mechanical properties and occurring deformation mechanisms of these immiscible polymer blends, is important with respect to potential mechanical recycling. This work focuses on the behavior of binary blends of linear low-density polyethylene (LLDPE), low-density polyethylene (LDPE), high-density polyethylene (HDPE), and polypropylene (PP) under tensile deformation and their related changes in crystallinity and morphology. All of these polymers plastically deform by shear yielding. When unmixed, the high crystalline polyolefins HDPE and PP both exhibit a progressive necking phenomenon. LDPE initiates a local neck before material failure, while LLDPE is characterized by a uniform deformation as well as clear strain hardening. LLDPE/LDPE and LLDPE/PP combinations both exhibit a clear-cut matrix switchover. Polymer blends LLDPE/LDPE, LDPE/HDPE, and LDPE/PP show transition forms with features of composing materials. Combining PP in an HDPE matrix causes a radical switch to brittle behavior.

## 1. Introduction

Plastics are ever-present in daily life situations. In 2018, 359 million tons were produced worldwide, and this annual amount continues to rise [1]. Of these produced plastics packaging is the predominant application, accounting for nearly 40% of total polymer demand [1]. In this sector, mostly commodity polymers such as (L) low-density polyethylene (LDPE), high-density polyethylene (HDPE), polypropylene (PP), polyethylene terephthalate (PET), polystyrene (PS), and polyamide (PA) are used. Because of the short lifetime of packaging products, these materials will end up in waste quickly, which causes them to be one of the main constituents of post-consumer plastic waste. Given that only 32.5% of collected post-consumer plastic waste is effectively recycled, the current research interest to improve both qualities and quantities of recycling is high [1].

One of the hurdles in recycling packaging is cross-contamination of one polymer by another, usually resulting in immiscible blends when processing the waste streams. As a result of this immiscibility the properties of the recycled blends will differ significantly from those of the virgin polymers [2]. In order to get high quality recycled products from plastic waste, it is important to separate and purify the waste streams as efficiently as possible [3]. Cross-contamination can occur in several situations. In the case of multilayer films, for example, polymers of different properties are combined in order to obtain a desired functionality (rigidity, barrier properties, printability, sealability, etc.) [4]. It is often not possible to separate the composing polymers as they are physically attached to one another and separation requires advanced techniques such as delamination whose cost often exceeds the value of the recycled product [5]. Another example can be found in the case of HDPE and PP bottles. These products are often composed of different (polyolefin) materials such as a label or a cap which are not removed in the sorting process and inevitably end up in the respective PP or HDPE sorted stream. Since the material densities of PP and HDPE are both lower than 1 g/cm^3^, a simple float-sink process is not feasible and in order to further purify this stream the recycler is forced to use alternative methods such as flake sorting using Hyperspectral Imaging by Near-infrared (NIR) or Raman [6].

Mechanical ‘as is’ recycling of these products will inevitably lead to the formation of immiscible blends. The goal of the current research is to quantify the effect of this unavoidable blending on the quality of the end stream, in order to determine the amount of pre-sorting, extra sorting, and purification steps necessary to achieve an acceptable quality. Miscibility, mechanical deformation, morphology, and crystallinity are the main factors which will influence the final properties of the recycled blends [2]. The deformation of mono semi-crystalline polymers was extensively investigated by Hiss et al. [7], Schrauwen et al. [8,9], and Pawlak and Galeski [10]. This paper examines the blending of four specific polyolefins: linear low-density polyethylene (LLDPE), low density polyethylene (LDPE), high density polyethylene (HDPE) and polypropylene (PP). Such blends are quite common in recycling practice, given how polyolefins are often separated from a mix by a sink-float process into the so-called mixed polyolefins (MPO) fraction, which is not further sorted.

As polyolefins are the most commonly used plastics, the amount of generic research conducted on the properties of blends of these materials is substantial. Typically, their mechanical properties are compared to the rule of mixtures, also known as the parallel model (Equation (1)). In this simple calculation, P is the investigated property of the blend, with P_i_ as the property of the pure polymers i (i = A or B) and x_i_ as the weight fraction of polymer i. The properties investigated within this study are modulus of elasticity, yield strength, strain at yield and strain at break.

(1) $$P=x_{A}\cdot P_{A}+x_{B}\cdot P_{B}$$

As regards to the combination of LLDPE with LDPE, Zhao et al. [11], Cho et al. [12], and Yamaguchi et al. [13] have done extensive work. They observed that mixing even these two related polymers will result in an immiscible blend [11]. Nonetheless, a yield strength larger than that predicted by the rule of mixtures was found [12,13]. Furthermore, a synergistic effect for strain at break over the whole composition range was detected [12]. Yamaguchi and co-workers performed a tensile test which saw an increase of the load until break [13].

Zhao et al. [11], Cho et al. [12], Hussein [14], and Gupta et al. [15] analyzed the LLDPE/HDPE blend. The miscibility of this blend depends mainly on the branching of the LLDPE. If the number of branches is too high, it was found that the mixture becomes immiscible. On the other hand, the length of these branches has no influence [11]. Furthermore, it is known that these polymers can co-crystallize [16,17]. The mechanical properties for this blend were found to be consistent with their composition according to the rule of mixtures. Strain at break also follows the rule of mixtures, as such blends with less than 60% HDPE are mainly influenced by the presence of LLDPE [12].

The final combination between the most common types of PE is LDPE/HDPE. This mixture has been analyzed by Zhao et al. [11], Cho et al. [12], Fu et al. [18], and Sarkhel et al. [19]. As with the LLDPE/HDPE blend, its miscibility depends on the branching of the polymers. If the LDPE has too many branches, the blend becomes immiscible [11]. Differential scanning calorimetry (DSC) measurements of LDPE/HDPE mixtures reveal two melting peaks, which indicates that the crystals of the two polymers are formed separately [12]. Hence, co-crystallization can only occur when the mixture is cooled down really fast [20]. Additionally, it was found that yield strength basically depends on the blend composition [12]. Furthermore Fu et al. [18] studied the point at which fibril formation begins after fragmentation of the lamellar crystals in a tensile test of different HDPE/LDPE blends. The blends all show the same stress–strain curves.

Li et al. [21], Strapasson et al. [22], Mofokeng et al. [23], Nolley et al. [24], Mastalygina et al. [25], and Tai et al. [26] have researched the mechanical properties of blends consisting of LDPE and PP. According to these studies, this blend is immiscible [21]. However, it was often found that yield strength and elastic modulus follow the rule of mixtures [22,23]. When looking at 50/50 wt.% compositions, this blend was found to display a brittle behavior [22] while for the 80/20 composition necking was observed [26]. Strain at break is a property which has not been considered much and when it was researched, no clear trend was observed [23].

LLDPE/PP has been researched by Li et al. [21,27] and Dumoulin et al. [28]. This blend has been reported as a miscible blend [21,27]. It has good results for deformations at low strain. When LLDPE is the majority phase, the elastic modulus has a negative deviation from the rule of mixtures. When the amount of PP is increased, the deviation becomes positive [28].

The last of these PE–PP blends, HDPE/PP, has been analyzed by Li et al. [27], Tai et al. [26], Niebergall et al. [29], Jose et al. [30], Lovinger et al. [31] and Finlay et al. [32]. The elastic modulus of this blend has been found to have a higher value in comparison to the rule of mixtures [29,31,32]. The other mechanical properties have a negative deviation from this rule [30]. It was found that yielding is accelerated when HDPE and PP are mixed and after yielding the material breaks almost immediately [31]. However, the elongation at yield was found to reach a maximum at the 60/40 HDPE/PP composition [29]. When the stress–strain curves were investigated, it was found that pure elements show necking and co-continuous blends (50/50) show brittle behavior. The intermediate compositions have matrix-drop dispersions and show a fluent transition stress–strain curve [32].

The adhesion between the semi-crystalline polymer interfaces of PP and different types of PEs has been investigated repeatedly [33,34,35,36,37]. The ability of PE to entangle and/or to epitaxially crystallize with the PP phase is important to obtain good adhesion. Polydispersity of the different blend constituents is found to be of crucial importance. During the synthesis of the polyolefins (metallocene vs. Ziegler–Natta catalysts), a low molecular amorphous fraction of chains can be formed. Poor adhesion between PE and PP can be ascribed to the diffusion of this oligomer content to the interface [34,35,36,37]. Processing is also important in obtaining strong interfaces, as a faster PE crystallization can result in an anchoring of the interfacial entanglements [33,35]. Jordan et al. [35] ascribed the brittle failure of Ziegler–Natta catalyzed HDPE and PP blends to this excess of oligomer at the interface. Entangled interfacial crystals in blends of metallocene catalyzed LLDPE and PP, which provide superior adhesion, resulted in ductile failure of their blends.

The method of processing will also have a major influence on the mechanical deformation of the materials investigated. Nolley et al. [24] investigated LDPE/PP blends made with compression molding and injection molding, in which the injection molded samples showed higher toughness. Godinho et al. [38] found higher strengths and stiffness for compression molded PE compared to injection molded test bars. This was attributed to the relatively slower cooling in compression molding, resulting in a more crystalline structure with larger spherulites. In Xie et al.’s study [39], however, higher elastic modulus and yield strength were obtained for injection molded ultra-high-molecular-weight polyethylene (UHMWPE)/PP blends, compared to the compression molding technique. This was explained by the skin–core structure caused by injecting and rapid cooling of the polymer melt. Here, there is a flow induced orientation of the dispersion phase, which consequently acts as a reinforcing element. Compression molded samples showed higher elongation at fracture and impact strength. This shows that the processing method influences the blend morphology, composition distribution and degree and type of crystallinity, and accordingly the mechanical properties of the polymer materials. In the current study, injection molding is chosen as a processing method for the test bars, conform ISO 527.

Much research has been done on the compatibility of polyolefins and how their properties change when they are mixed. However, typically such research is conducted on a single blend combination—often even for just one composition ratio—and limited to quantitative description of observed effects. As these studies give information on various combinations with different grades of polyolefins every time, it is nigh impossible to make a valid comparison between all the binary polyolefin blends’ behaviors. Moreover, the existing literature often details observed values for mechanical properties like modulus or strength, but rarely details the deformation mechanism which occurs. This paper aims to fill this gap methodically. It examines the changes in mechanical properties and deformation mechanisms of polyolefins in combination with other polyolefins (PO–PO) for all binary combinations of LLDPE, LDPE, HDPE, and PP, with compositions of 95/5, 90/10, 80/20, and 50/50 for each blend as well as the pure polymers.

## 2. Materials and Methods

### 2.1. Materials

Four polyolefins were used in this work: LLDPE, LDPE, HDPE, and PP. Their respective grades, producers and melt flow indices (MFI) can be found in Table 1. The LLDPE material is an ethylene 1-hexene copolymer.

### 2.2. Sample Preparation

For this study each polymer listed in Table 1 was physically blended with each one of the other polymers by hand mixing (such physical blending was chosen to represent the typical mechanical recycling process in industry). The whole composition was to be investigated, so the following compositions were prepared: 0–5–10–20–50 wt.% polymer A in polymer B, with all four polymers being used for both A and B.

These blends were processed by injection molding using an Engel 28-ton injection machine (Engel e-victory, Schwertberg, Austria) into ISO 527-2/1A tensile dog bones. The temperature profile used for all PEs was, from hopper to nozzle, 160C–170 °C–180 °C–190 °C. The blends which included PP had a profile going from: 200 °C–210 °C–220 °C–230 °C. The mold temperature was the same for all blends, namely 15 °C. All other parameters can be found in Supplementary Materials.

### 2.3. Mechanical Characterization

The tensile test (ISO 527-2) was performed with an Instron 5565 tensile testing machine (Norwood, MA, USA). A load cell of 5 kN was used. The dynamometer was equipped with an Instron clip-on extensometer for an accurate determination of the modulus. A pre-load of 40 or 60 N was set, depending on the sample. First, a speed of 1 mm/min was used for all blends. Depending on the sample, the speed was raised to 50 or 75 mm/min after reaching a strain of 0.3%. Out of the tensile test, elasticity modulus (E), yield strength (σ_y_), strain at yield (ε_y_) and strain at break (ε_b_) were calculated. For LLDPE and LDPE (pure or as the majority component) σ_y_ was determined by the 0.2% offset method. The σ_y_ of HDPE and PP (pure or as the majority phase) was analyzed as the zero-slope yield strength. The strain corresponding to σ_y_ is ε_y_.

### 2.4. Differential Scanning Calorimetry (DSC)

DSC measurements were performed on a DSC 214 Polyma of Netzsch (Selb, Germany). Two cycles of heating and cooling were executed starting from 30 °C and going up to 300 °C, with a heating rate of 10 K/min. In between the dynamic heating/cooling cycles an isothermal of 5 min was introduced. Crystallinity was determined in the first run using Equation (2), where $\Delta H_{m}$ and $\Delta H_{cc}$ are the enthalpy of melt and cold crystallization of the polymer respectively. These values are the average of two measurements. $\Delta H_{m}^{\infty }$ is the theoretical enthalpy of 100% crystallization of the polymer and x is the weight fraction of the polymer in the blend. The first run was selected to determine how the samples are mixed after processing and how this affects the properties.

(2) $$X_{c}\left(\%\right)=\frac{\Delta H_{m}-\Delta H_{cc}}{\Delta H_{m}^{\infty }\cdot x}\cdot 100\%$$

When there was an overlap of the peaks, the software ‘Peak Separation 3’ (Netzsch) was used to make a prediction of each peak separately. The general prediction formula was used, which is a weighted mixture of Fraser–Suzuki and asymmetric Cauchy. When a prediction was not possible for strongly overlapping peaks, the total crystallinity is reported.

For all materials a prediction is calculated for the crystallinity in all blends. This prediction uses the rule of mixtures (Equation (1)), which is known to be valid for physical properties like density and X_c_. P_A_ and P_B_ are the crystallinities measured for the mono polymers.

### 2.5. Scanning Electron Microscopy (SEM)

To investigate the morphology of the blends, non-deformed injection molded samples were cryogenically fractured in liquid nitrogen. All these samples were sputtered with a gold coating using BAL TEC SCD 005 sputter coater with 25 mA for 40 s (Balzers, Liechtenstein). Micrographs were made with a JEOL JSM 7600F scanning electron microscope (Tokyo, Japan). The accelerating voltage used was 20 kV. Note that the images shown below have different magnifications, as their differences in structure are best displayed at the magnifications used here.

## 3. Results

### 3.1. Morphology of Blends

In Table 2, different SEM-images are depicted for LLDPE + PP, LDPE + PP, and HDPE + PP. It was not possible to detect the morphology of any of the PE + PE combinations because of their similar response to the electron beams. The mixtures with LDPE display a veined structure for 20 and 50 wt.% PP as can be seen in Table 2. LDPE deformed plastically during preparation of SEM (cryogenically broken). Cryogenic breaking of the HDPE phase led to a partially ductile fracture, which can be seen as the white small dots on the micrographs (Table 2) [40].

All 80/20 and 20/80 combinations have a droplet-matrix morphology. The matrix is formed by the polymer in excess. With the exception of 20/80 LLDPE/PP, none of these mixtures exhibits a uniform shape of droplets. A difference in morphology was observed between the center of the part and the exterior of the part (closer to the mold surface). In the outer region of the test bars, a more fibril-like structure was observed, which can be ascribed to the high shear during injection molding.

Another criterion that can be evaluated in these micrographs is the apparent adhesion between phases. When two phases display a good interaction, no detachment of the droplet particles from the matrix is observed. This is the case for HDPE and PP blends. The adhesion between phases deteriorates with LLDPE or LDPE in combination with PP. In the case of LDPE + PP the detachment of the droplet particles from the matrix is more pronounced. The blend of LLDPE with PP shows the worst adhesion of the whole series. A clear black interface can be noticed, indicating detachment between the phases.

In general, a co-continuous structure is observed for 50/50 polymer blends. A mixture can only be called co-continuous when both materials have a 3D continuity over the whole sample [41]. HDPE + PP and LDPE + PP fulfil this definition. For LLDPE + PP, the 50/50 composition still exhibits a droplet-matrix morphology.

### 3.2. Crystallinity

#### 3.2.1. Mono Materials

The crystallinity of the mono materials can be found in Table 3. LLDPE and LDPE have a relatively low crystallinity, 28.8 and 32.7% respectively, in comparison to HDPE and PP, which have a crystallinity of 65.1 and 42.2% respectively. Upon changing the processing temperatures, no noteworthy changes in crystallinity were observed.

#### 3.2.2. Blends

**Figure 1:**
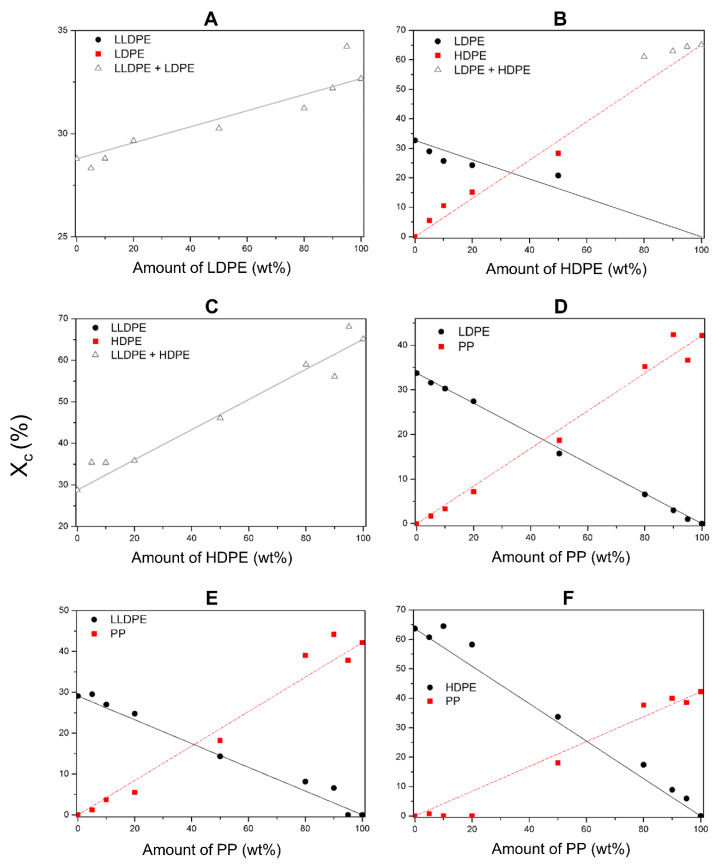
Crystallinity of all blends: (**A**) LLDPE + LDPE; (**B**) LDPE + HDPE; (**C**) LLDPE + HDPE; (**D**) LDPE + PP; (**E**) LLDPE + PP; (**F**) PP + HDPE. The symbols represent the experimental measured values. Lines present the predictions (____: prediction of Δ, ____: prediction of ●, _ _ _: prediction of ■). The predictions are calculated using the rule of mixtures (Equation (1)), based on the crystallinities of the mono materials. The data points are the averages of two measurements.

The crystallinity of all discussed blends are depicted in Figure 1, which shows both the experimental data and a predicted value according to Equation (1). In mixtures LLDPE + LDPE and LLDPE + HDPE, an overlap of the melting peaks was observed in DSC measurements which could not be deconvoluted. The graphs of these materials show the total crystallinity. For LLDPE + LDPE the crystallinity remains almost constant (Figure 1A). These materials do not have an influence on each other during crystallization. The total crystallinity of LLDPE + HDPE mixtures decreases, while the amount of LLDPE increases (Figure 1C). Within the mixture HDPE + LDPE, peak separation was not possible for the mixtures with HDPE as the majority component (Figure 1B). When LDPE is the majority phase, its crystallinity is lower than predicted, while for HDPE an increase is noticed. This means that adding HDPE to LDPE has a negative influence on the crystallinity of LDPE. However, the matrix LDPE has a positive influence on the crystallinity of the dispersed HDPE phase.

When PP droplets are present in a LLDPE matrix, the crystallinity of PP is lower than predicted and LLDPE follows the trend of the prediction (Figure 1E). However, when PP is the matrix, both materials have a higher crystallinity than predicted. These materials have a positive influence on each other’s ability to crystallize. In the LDPE + PP blends the crystallinity values follow the prediction, with the exception of 10 wt.% LDPE in PP (Figure 1D). When PP is present as droplets in a HDPE matrix, the material hardly crystallizes at all (Figure 1F). On the other side of the composition range for the HDPE + PP blends, in which PP constitutes the matrix, the crystallinity of HDPE is higher than predicted and the crystallinity of PP follows the prediction.

### 3.3. Tensile Deformation and Properties

#### 3.3.1. Mono Materials

**Figure 2:**
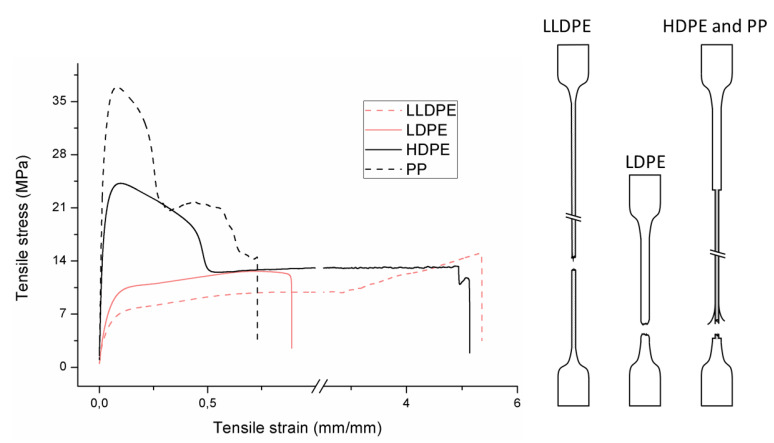
Stress–strain curves of the mono materials with schematic representation of the deformed samples.

The tensile properties can be found in Table 3 and Figure 2. Their crystallinity was considered an important factor in interpreting the results. As can be deduced from Figure 2, HDPE and PP display typical shear yielding deformation and LLDPE and LDPE do not. However, HDPE and LLDPE both have a high ε_b_.

When examining the deformed samples, it can be seen that PP and HDPE both display fibrillation on the outside of the sample and undergo a progressive necking phenomenon (Figure 2). Images of this macroscopic fibrillation can be found in Supplementary Information. Therefore, both polymers were classified as high crystalline materials with neck shear yielding.

There is a clear difference between how LDPE and LLDPE deform. LDPE initiates very localized necking which does not propagate before failure takes place (Figure 2) and hereafter we refer to local shear yielding. Furthermore, it can be seen in the tensile curve that LLDPE undergoes strain hardening, which LDPE does not. Thereby a large ε_b_ (427%–557%) can be observed for LLDPE. Moreover, the cross section of the LLDPE material becomes smaller over the whole tensile test bar during the tensile test, and fibrillation does not occur (Figure 2). Hereafter, we refer to uniform shear yielding with strain hardening.

#### 3.3.2. Blends

**Figure 3:**
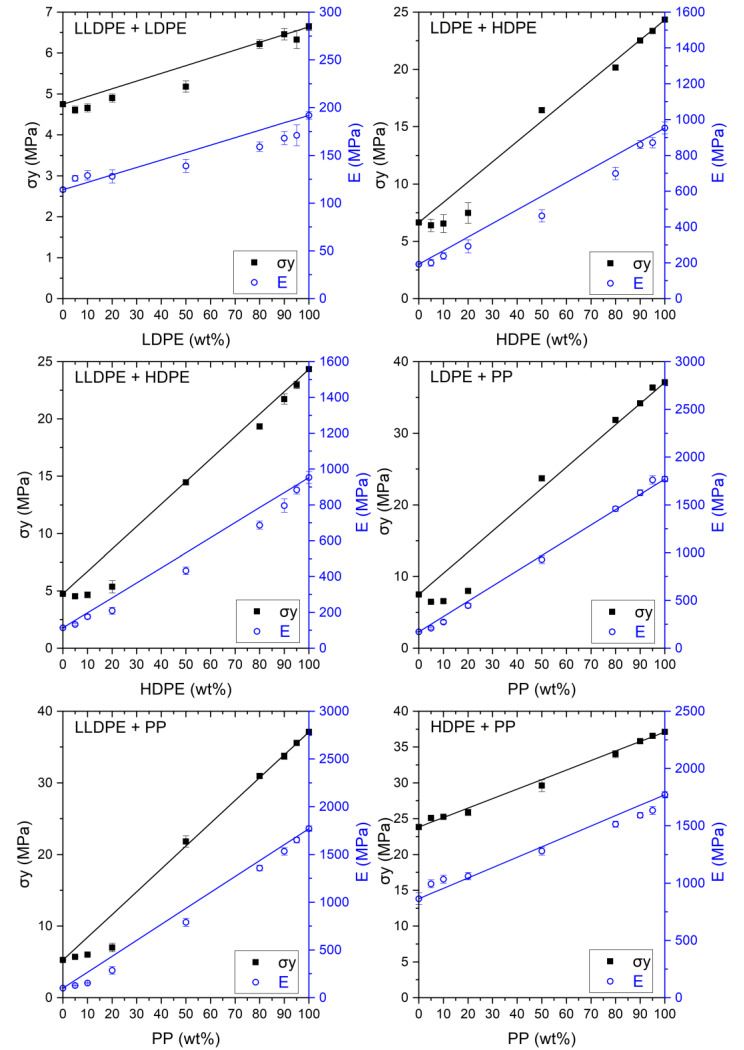
Overview of the σ_y_ and E values for the investigated blends: LLDPE + LDPE; LDPE + HDPE; LLDPE + HDPE; LDPE + PP; LLDPE + PP; HDPE + PP. Lines give the prediction based on the rule of mixtures for σ_y_ (black) and E (blue).

The blends were analyzed for their deformation mechanism and tensile properties. The E and σ_y_ properties are presented in Figure 3. All deformation mechanisms were divided into six types. Table 4 includes the typical stress–strain curves and test sample deformations divided by type. Types A, B, and C are for ‘HDPE and PP’ (A), LDPE (B), and LLDPE (C), respectively. The types AB, AC, and AA are explained in more detail below but in essence they describe transition deformations from the one type (first letter) to the other (second letter). Table 4 is related to Table 5, which shows how each blend deforms. These two tables are the basis for the description which follows.

##### LLDPE + LDPE

When combining LDPE and LLDPE, no transition form is observed, as can been seen in Table 5. However, there is a switchover of the matrix. At the 50/50 composition the deformation is like mono LDPE. When the amount of LLDPE increases, ε_b_ becomes larger and the samples after deformation became thinner over the whole sample. A smooth transition in deformation mechanism from LDPE (Table 4B) towards the mechanism of LLDPE (Table 4C) is noticed when LLDPE is added to LDPE. E and σ_y_ slowly increase when LDPE is added. When these properties are compared to the rule of mixtures, the values of E and σ_y_ are within a 10% range of the predicted values.

##### HDPE + PP

These materials normally deform in a similar ductile manner, but when PP is added to HDPE (including 50/50 composition) brittle behavior is observed (Table 4 curve AA and Table 5) and as a result, ε_b_ drops extremely. On the other side of the composition range (adding HDPE to PP) this brittle behavior is not observed and an increase of ε_b_ is even noted. These blends continue to deform like pure HDPE (Table 4, curve A). The yield strength (σ_y_) and modulus (E) of the blends follow the rule of mixtures.

##### LLDPE + HDPE

When combining LLDPE and HDPE, the deformation characteristics of both materials are evident. The 50/50 and 80/20 HDPE/LLDPE blends have a tensile curve type AC (Table 4). In the first part of the tensile test, the material deforms by the shear yielding mechanism, the same as pure HDPE. Necking was also observed in the deformed samples, but here the cross section was bigger than with pure HDPE. In the second part strain hardening occurs, which is clearly a phenomenon of LLDPE, which we refer to as neck shear yielding with strain hardening. E increases linearly when HDPE is added to LLDPE. As long as LLDPE is the majority component, σ_y_ remains constant. For blends of 0–50 wt.%, σ_y_ follows the rule of mixtures. ε_b_ decreases until 50 wt.% HDPE when it is added to LLDPE. Adding LLDPE to HDPE gives an increase in ε_b_, except for 5 wt.% LLDPE.

##### LLDPE + PP

The blend LLDPE + PP blend has no transition form within the tested range. There is a clear switchover of the matrix. At the 50/50 composition the material deforms like the pure PP (Table 5) even though the E rises linearly. When LLDPE is the majority component, σ_y_ remains constant until 20 wt.% PP and increases thereafter. When PP is the majority phase, σ_y_ drops linearly. ε_b_ increases on both sides of the composition range, reaching a maximum at 50/50 LLDPE/PP.

##### LDPE + HDPE

This blend has a transition form (Table 5) at the 50/50 composition, namely combined (neck + local) shear yielding (Table 4, curve AB). The deformed samples have more constriction, but the necking phenomenon from HDPE does not take place. For other weight ratios, the blend deforms, behaving like the polymer in excess, which means that the necking phenomenon takes place for HDPE as the majority phase. HDPE is responsible for the increase of E and σ_y_. LDPE is responsible for the decrease of ε_b_ in this mixture.

##### LDPE + PP

When LDPE or PP is the majority component, the blend deforms like the polymer in excess. When PP is the matrix, multiple constrictions are observed, where the sample breaks at the weakest spot. For the 50/50 composition this blend has the transition form of combined (neck + local) shear yielding (Table 4, curve AB, Table 5). For this composition the necking phenomenon of PP does not take place, but the samples have more constriction than with LDPE as matrix. PP is responsible for the increase of E and σ_y_. The lowest value for ε_b_ is observed for the 50/50 composition. Addition of LDPE to PP gives an increase of ε_b_, while addition of PP to LDPE ε_b_ remains constant.

## 4. Discussion

### 4.1. LLDPE + LDPE

**Figure 4:**
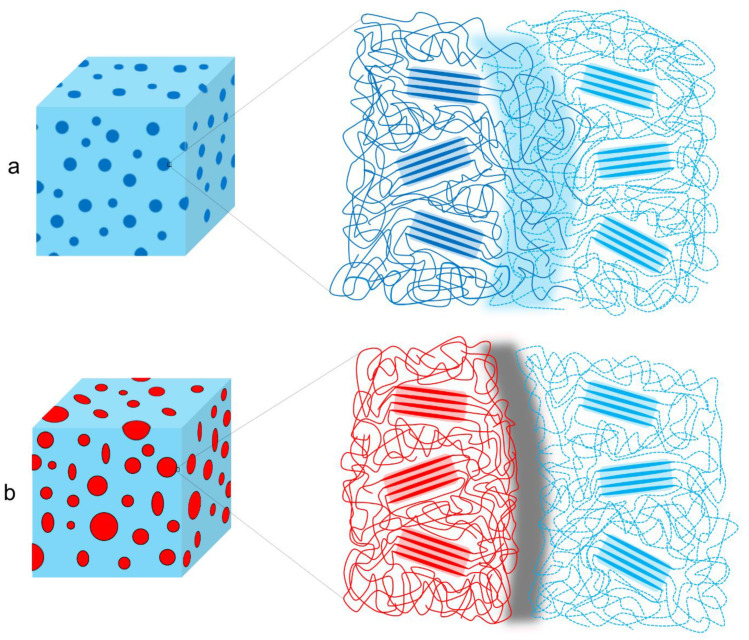
Miscibility at the polymer blend interphase: (**a**) compatible polymer blends with a mixed amorphous interphase and (**b**) immiscible polymer blends with clear phase separation.

LLDPE and LDPE have the same monomer repeating unit, which should result in a good miscible mixture. This was confirmed by Cho et al. [12], who found that the materials are miscible in the amorphous phase. Visual examination of this mixture through SEM was not practically possible because these materials have almost the same density so that the contrast in SEM is insufficient. Instead, some assumptions can be made in terms of morphology. When one of the two materials is in excess, a droplet-matrix morphology with small droplets and a mixed amorphous phase at the interphase can be assumed, due to the good miscibility of both polymers. For the 50/50 composition, a co-continuous morphology of LLDPE and LDPE phases is presumed. This is supported by the fact that equal amounts of both polymers are present, their similarity in rheological behavior and the empirical verification (by SEM analysis) of a co-continuous structure in the LDPE + PP and HDPE + PP blends discussed in the results section. A mixed amorphous interphase (Figure 4a) is expected to be present, meaning that the chains of both polymers in the blend are mixed in the amorphous phase, resulting in a stronger interphase.

The two materials have different chain structures. LDPE has short and long branches which themselves contain side branches. LLDPE is known for its large number of short and uniform branches. Though its branches are shorter, there are more of them and they are able to move against one another upon elongation without entangling. Furthermore, LLDPE forms looser crystals, because the short branches disturb the crystals which lead to less dense folds of the lamellae [42]. As a result, slip of the chains will occur more easily and the chains can align, which explains the strain hardening of LLDPE.

On the other hand, LDPE’s longer branches easily get entangled. Due to this branched structure of LDPE the total deformation of the material is limited. The entanglements between the branches will lead to high stresses and failure will occur.

With an increasing amount of LLDPE the ε_b_ increases over the whole composition range, which is not in compliance with the rule of mixtures. Due to the presence of LDPE, LLDPE cannot align as well and the strain hardening effect will decline. At the 50/50 composition ε_b_ amounts the average of the mono materials. In this co-continuous morphology, the phases are finely distributed through the whole sample. This results in a large interphase. At the interphase LDPE is less entangled because the presence of the linear LLDPE, partly hinders this process. Therefore, LDPE can align more than mono LDPE, resulting in more slip. For LLDPE, however, the presence of LDPE results in fewer possibilities for stretching and strain hardening will not be visible. When LDPE forms the matrix, ε_b_ increases with rising amounts of LLDPE, although this increase is limited. However, the presence of LLDPE decreases the possibility of entanglements whereby ε_b_ increases. Furthermore, the increasing ε_b_ for higher amounts of LLDPE can be ascribed to the stronger interphase that is formed because less oligomer content is present. This amorphous fraction of short chains is responsible for poor adhesion at the polyolefin interface, as described by Jordan et al. [35], Lo et al. [36], and Poon et al. [34]

For this mixture E and σ_y_ follow the rule of mixtures within a 10% range. Yamaguchi et al. [8] claimed that the increase of σ_y_ with an increasing amount of LDPE is a result of an increasing total crystallinity, which is also the case here. In fact, the total crystallinity also follows the rule of mixtures within a 5% range. Therefore, it can be assumed the materials do not disturb each other’s crystallinity. It can be concluded that these materials follow the rule of mixtures because of their good miscibility in the mixed amorphous interphase.

### 4.2. HDPE + PP

When examining the morphology of PP in HDPE (Table 2), one observes that PP forms larger droplets in HDPE than the other way around. This is a direct result in difference of flow behavior. HDPE has a lower viscosity than PP, so that HDPE phases are more likely to break up into smaller particles than PP. At the interphase there is no mixed amorphous phase (Figure 4b) because these materials are not miscible [21]. Larger droplets make the possibility of cracks forming more likely, which eventually leads to brittle behavior through propagation of these cracks [43]. This explains the brittle behavior of HDPE with 5–20 wt.% PP regardless of the good adhesion which can be seen in microscopy (Table 2). A co-continuous morphology was observed for the 50/50 composition (Table 2), which also displayed brittle behavior because of the large interphase between the two materials. This brittle behavior (for 5–50 wt.% PP) is a result of the high crystallinity of both materials which lead to high local stresses in combination with an easy propagation of cracks.

When PP is the matrix, the addition of HDPE leads to an increase of ε_b_. Lack of debonding indicates good adhesion, which is visible between the materials with microscopy (Table 2). Blom et al. [44] claim there is a certain degree of interaction between HDPE and PP for concentrations of HDPE below 20 wt.%. PP has a higher σ_y_ than HDPE, therefore we suppose an isostrain deformation of the blend, at least up to yielding. HDPE will start plastic deformation while PP is still deforming elastically, which results in elongated HDPE particles. This eventually results in a toughening of the blend.

E follows the rule of mixtures within a 10% range. Lovinger et al. [31] claim that for small strain the influence of incompatibility is negligible but that the effect of tie chains and intercrystalline links are significant. These tie chains and intercrystalline links transmit stress between the lamellae of the materials. For the yield strength they observed the same trend, namely σ_y_ follows the rule of mixtures (within a 5% range) [31], which implies a good adhesion between the phases.

As regards the crystallinity, there is a difference between matrix HDPE and PP. Both materials have a high crystallinity 65% and 42%, respectively. When HDPE forms the matrix, PP has a low crystallinity, more than 20% lower than the prediction by rule of mixtures, because of a lower nucleation density. The crystallinity of HDPE is higher than the prediction as a result of the possibility of nucleation on the PP phase. When PP is the majority component, the crystallinity of PP remains constant and is a nucleator for HDPE, which results in a higher HDPE crystallinity. Li et al. [21] confirm this heterogeneous nucleation.

### 4.3. LLDPE + HDPE

The morphology of the LLDPE + HDPE blend could not be observed for the same reason as that of LLDPE + LDPE. However there are TEM images of HDPE/very low-density polyethylene (VLDPE) 80/20 to be found in the literature [43] that could be considered valid for the morphology of this blend as the structure of LLDPE is very similar that of VLDPE. A droplet-matrix morphology was noticed and at the interphase the lamella of HDPE diffused in the VLDPE phase. For the 50/50 composition a co-continuous morphology is assumed based on the findings for other blends discussed in this work. We assume that this blend also contains an interphase with a good miscible amorphous phase. Note that this interphase is not as strong as the mixed interphase in LDPE/LLDPE blends because of fewer possibilities for entanglement. Due to its more linear structure HDPE and LLDPE will align more easily than mixtures with LDPE and eventually positively effects ε_b_.

HDPE and LLDPE are both linear structures and have the same monomer repeating unit. The difference in structure is the number of short branches on the backbone. HDPE has fewer branches and therefore forms strong large crystals. When the material is stretched, a local weakness will lead to the start of necking before the crystals can deform. LLDPE has more short branches which lead to smaller looser crystals, as noted above. Hereby the material will deform more uniformly over the whole sample because of the multiple small crystals. This was observed from the deformed samples after tensile test. In the blends of these materials the crystallinity changes with a maximum deviation of 15% from the rule of mixtures because the materials do not have an influence on each other (Figure 1).

E increases when the amount of LLDPE decreases [14] because LLDPE has a lower E than HDPE. Because of the above—noted negligible incompatibility at low strain [31]—both materials play an important role in the determination of E. When the blends are composed of an LLDPE matrix with an HDPE dispersed phase, σ_y_ remains constant over the different compositions. LLDPE yields earlier than HDPE, therefore it determines the yield of the blend when it is the matrix. When HDPE is the matrix, σ_y_ follows the rule of mixtures because less HDPE is present to carry the overall load.

When HDPE is added to an LLDPE matrix, ε_b_ decreases. HDPE acts as stiff particles and disturbs the ability of LLDPE to align its chains. Therefore, the strain hardening will become less pronounced. For the blend 5/95 LLDPE/HDPE there is a decrease of ε_b_ compared to the mono HDPE. The deformation is determined by the matrix. There is not enough LLDPE present to disturb the crystal size, and the deformation will stay the same. For 10 and 20 wt.% LLDPE, ε_b_ is higher than mono LLDPE and mono HDPE. HDPE will form smaller crystals as a result of the presence of LLDPE. As a result, there is a more uniform deformation and the whole sample contributes to dividing the stresses, so that higher ε_b_ can be reached. This phenomenon can also be seen for the 50/50 composition. Strain hardening of LLDPE is still visible at 20 wt.% LLDPE in HDPE. Both materials have a linear structure so that they align more easily together, leading to strain hardening of the LLDPE material.

### 4.4. LLDPE + PP

For LLDPE + PP there is no mixed amorphous phase at the interphase because of the difference in monomer repeating unit (Figure 4b). At 50/50 composition, this blend deforms like the mono PP. Morphology (Table 2) confirms PP as the matrix for the 50/50 composition, whereby it determines the deformation. There is also another explanation for the absence of the strain hardening of LLDPE for the 50/50 blend. LLDPE does not have the possibility of deforming by strain hardening before the PP phase fails. When the PP phase fails, there is higher stress on the LLDPE phase, which results in complete failure before strain hardening is noticeable. For this blend co-continuous morphology was not observed because the range of co-continuous blend formation was not analyzed in this work.

The E increases when the amount of LLDPE decreases [14] because LLDPE has a lower E than PP. Both materials contribute to the stiffness of the blend because E is measured at low strain levels [31]. However, E does not follow the rule of mixtures, which can be linked to the crystallinity (Figure 1). PP as the majority component results in a higher crystallinity for both LLDPE and PP whereby E remains high. When the amount of LLDPE increases, the deviation from the rule of mixtures increases. When LLDPE forms the matrix, the crystallinity of PP is lower than predicted because of interference on crystallization due to a low nucleation density [2]. The crystallinity of LLDPE follows the prediction when it forms the matrix and as a result, E is lower than the rule of mixtures with a deviation greater than 20% from the prediction.

When LLDPE is the matrix, σ_y_ remains constant. The distribution of PP in LLDPE is not uniform (Table 2) so that LLDPE determines the deformation of the blend. This is a result of the different flow behavior of the materials. When PP is the matrix, σ_y_ follows the rule of mixtures. The contribution of LLDPE and PP is equal because σ_y_ is still measured at low strain levels, as E and the distribution of LLDPE in the PP matrix is more uniform.

LLDPE has a toughening effect on PP and postpones fibrillation [15]. In the PP + LLDPE blend there is a constant increase of. LLDPE acts as an elastomer in the PP matrix.

### 4.5. LDPE + HDPE

As it is another binary blend of PEs, SEM imaging of this blend was not possible. Zhao et al. [11] discuss the miscibility of PE blends and conclude that LDPE and HDPE are not miscible, except for a number of branches lower than 20/1000 backbone C’s. This immiscibility will lead to relatively little adhesion between these materials.

E increases as the amount of HDPE increases, within a 20% range of the rule of mixtures. This can be linked to the amount of crystallinity, which increases with the amount of HDPE. If LDPE is the matrix, σ_y_ remains constant because the deformation is determined by the LDPE matrix. From 50 wt.% HDPE onwards, σ_y_ follows the rule of mixtures. HDPE is the stronger material which becomes weaker by adding LDPE, because less HDPE is present in the blend to carry the load.

An LDPE rich blend deforms as mono LDPE and HDPE has no influence because the adhesion is expected to be low. ε_b_ remains almost constant when LDPE constitutes the matrix. For the 50/50 composition there is an assumption of a co-continuous morphology. The two materials will stretch together and HDPE will initiate necking. At a certain point the LDPE phase will reach its maximum strain and will break, so that the whole sample will break. When a matrix HDPE is present, LDPE will decrease ε_b_ because less HDPE is present to perform the shear yielding with a long stable necking phenomenon. The typically lower levels of adhesion can also be attributed to the higher amounts of non-crystallizable short chains, due to the increasing content of LDPE. This amorphous fraction can diffuse to the interphase and result in lower ε_b_ values [34,35,36].

### 4.6. LDPE + PP

The LDPE + PP blend is not miscible [21], so that we can assume it does not have mixed amorphous interphase (Figure 4b). The 50/50 composition has a co-continuous morphology (Table 2). Therefore, the properties of both pure materials will be evident. By adding LDPE to a PP matrix, the necking phenomenon is postponed. When 50 wt.% of LDPE is reached, the sample breaks before necking occurs (a reduction in cross section was visible before breaking). This is a result of the failing of the LDPE material because the maximum load which LDPE can handle is surpassed, which results in higher stress on the remaining PP phase.

E increases when the amount of LDPE decreases [26], following the rule of mixtures. Both materials contribute as this property is measured at low strain level [31]. If LDPE is the matrix, σ_y_ remains constant. From 50/50 composition onwards σ_y_ increases with the rule of mixtures. This is similar to LDPE + HDPE. As LDPE is the matrix the deformation is determined by LDPE and as PP forms the matrix it is weakened by the addition of LDPE.

Strain at break varies with the composition. The 50/50 LDPE/PP blend is co-continuous and results in the lowest ε_b_ determined by the LDPE phase. When LDPE forms the matrix, it determines ε_b_ and the deformation so that ε_b_ remains constant. When PP is the majority component, ε_b_ decreases with an increasing amount of LDPE. The presence of LDPE dispersed phase helps to divide the internal stresses of the PP matrix whereby the cross section of the deformed sample increases, but less PP is present to perform shear yielding with a stable necking phenomenon.

In this blend there is heterogenic nucleation of the materials [21]. When LDPE is the matrix, the crystallinity of PP is lower than the prediction because LDPE inhibits the spherulite growth of PP. When PP is the matrix, the crystallinity of LDPE is lower than the prediction because PP is already solidified when LDPE so that it hinders the crystallization.

## 5. Conclusions

This paper has focused on the link between the mechanical properties and the deformation mechanisms which take place during tensile loading of binary polyolefin–polyolefin blends. Four polyolefins (LDPE, LLDPE, HDPE, and PP) were blended and processed, covering the whole composition range.

Morphological analysis showed that phase separation occurs when processing these polymers as blends, despite their basic structural similarities. The mixtures are characterized by—depending on the rheology and processing characteristics of the polyolefins—a droplet-matrix morphology, which shifts towards a co-continuous structure in the range of equal mass ratios.

The pure polyolefins each exhibit their own signature tensile stress–strain behavior and corresponding deformation mechanism. The two high crystalline polymers HDPE and PP undergo shear yielding and display clear neck formation. The low crystalline polymers LDPE and LLDPE are characterized by the distinct deformation mechanisms local shear yielding in the former, and uniform shear yielding with strain hardening in the latter.

In the polymer blends, apart from their morphology and crystallinity, their miscibility and related adhesion at the interphase are crucial in their mechanical characteristics. The combinations LLDPE/LDPE and LLDPE/HDPE are assumed to have a strong interphase due to the miscibility of the amorphous phases of both polymers.

LLDPE/LDPE and LLDPE/PP blends do not show a transition form in deformation mechanism, but a clear matrix switch-over. The polymer blends LDPE/HDPE, LDPE/PP, and LLDPE/HDPE exhibit transition tensile deformations, in which the characterizing deformation mechanism of both pure polymers emerge. Blending PP into HDPE leads to brittle structures.

These insights into mechanical deformation mechanisms help to build up our knowledge of the effects of cross contaminations between polymers, the resulting (change in) mechanical properties and, ultimately, their relative value for recycling.

## Figures and Tables

**Table 1:** Used materials.

Material	Grade	Producer	MFI (g/10 min) *
LLDPE	Exceed^TM^ 1012HA	ExxonMobil	1.0
LDPE	LD150AC	ExxonMobil	0.8
HDPE	25055E	Dow	25.0
PP	PP6272NE1	ExxonMobil	2.8

* MFI—PE: 190 °C—2.16 kg and PP: 230 °C—2.16 kg.

**Table 2:** Scanning electron microscopy (SEM) images of the 80/20, 50/50, and 20/80 ratios for the binary blends of PP mixed with LLDPE, LDPE and HDPE. LLDPE + PP: droplet/matrix morphology for all three ratios; LDPE + PP: 80/20 and 50/50 shows plastic deformation of the LDPE phase in contrast to the PP phase; HDPE + PP: 80/20 and 50/50 ductile fracture of the HDPE phase.

Blend (A + B)	Composition (A/B)		
	80/20	50/50	20/80
LLDPE + PP	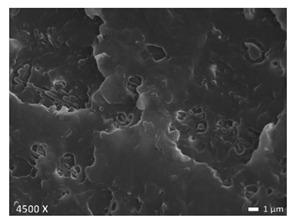	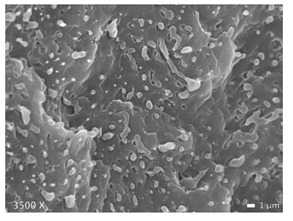	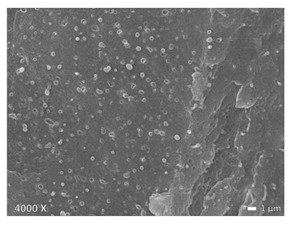
LDPE + PP	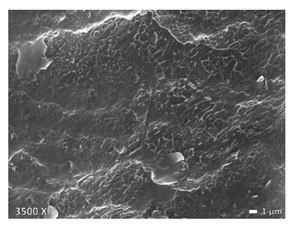	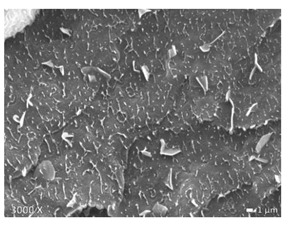	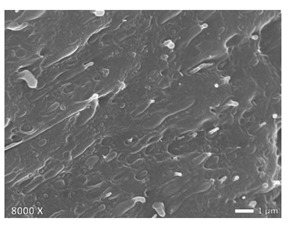
HDPE + PP	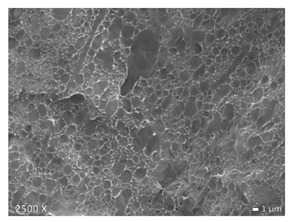	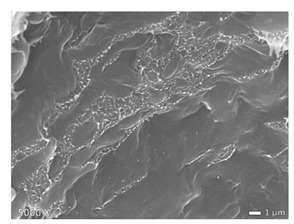	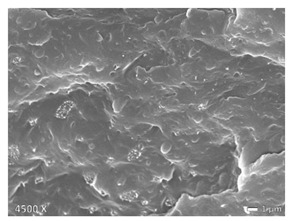

**Table 3:** Properties of used mono materials.

Material	T_processing_ (°C)	X_c_ (%) ^1^	E (MPa) ^2^			σ_y_ (MPa) ^2^			ε_y_ (MPa) ^2^			ε_b_ (%) ^2^		
LLDPE	190	28.8	114	±	2	4.75	±	0.05	3.41	±	0.07	557	±	12
	230	29.1	101	±	2	5.26	±	0.03	3.79	±	0.08	427	±	23
LDPE	190	32.7	192	±	4	6.64	±	0.09	3.08	±	0.09	89.0	±	2.0
	230	33.8	172	±	4	7.51	±	0.10	3.57	±	0.10	114	±	3
HDPE	190	65.1	953	±	33	24.34	±	0.15	9.77	±	0.08	463	±	110
	230	63.7	864	±	51	23.83	±	0.38	10.62	±	0.14	269	±	57
PP	230	42.2	1771	±	28	37.11	±	0.17	8.09	±	0.06	68	±	9

^1^ Values based on the average of two measurements.^2^ Tensile test was performed 10 times and the average values and standard deviations are reported here.

**Table 4:** Stress–strain curves with a representative code, schematic dog bone deformation, mechanism, and typical materials.

Code	Curve	Test Bar Deformation	Deformation Mechanism Polymers/Blends
A	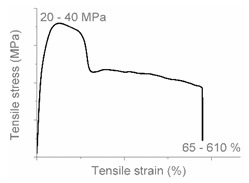	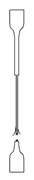	Neck shear yielding HDPE (pure, ε_b_: 270%–465%) PP (pure, ε_b_: 70%)
B	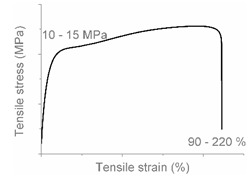	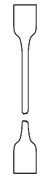	Local shear yielding LDPE (pure, ε_b_: 90%–115%)
C	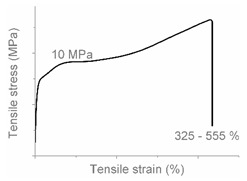	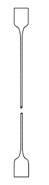	Uniform shear yielding with strain hardening LLDPE (pure, ε_b_: 430%–555%)
AB	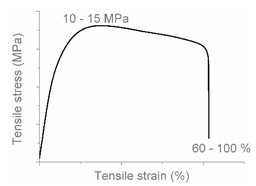	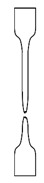	Combined (neck + local) shear yielding LDPE + HDPE LDPE + PP
AC	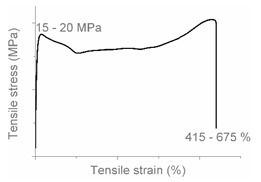	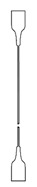	Progressive shear yielding with strain hardening LLDPE + HDPE
AA	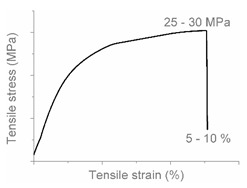	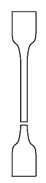	Brittle HDPE + PP

**Table 5:** Typical deformation curve for each blend, the letter of which corresponds to the deformation mechanisms described in Table 4.

Amount of A (wt.%)	A	B		
		LLDPE	LDPE	HDPE
0	**LDPE**	C		
5		C		
10		C		
20		C		
50		B		
80		B		
90		B		
95		B		
100		B		
0	**HDPE**	C	B	
5		C	B	
10		C	B	
20		C	B	
50		AC	AB	
80		AC	A	
90		A	A	
95		A	A	
100		A	A	
0	**PP**	C	B	A
5		C	B	AA
10		C	B	AA
20		C	B	AA
50		A	AB	AA
80		A	A	A
90		A	A	A
95		A	A	A
100		A	A	A

## Supplementary Materials

The following are available online at https://www.mdpi.com/2073-4360/12/5/1171/s1, Tables including Injection molding parameters; mechanical properties of the investigated blends; DSC results of the investigated blends. Figures including DSC curves; pictures of the tested samples.
