# Supplementary material for: Microstructural Contributions of Different Polyolefins to the Deformation Mechanisms of Their Binary Blends
Source: Polymers (Basel). 2020 May 20;12(5):1171. doi: 10.3390/polym12051171 (PMC7285311; doi:10.3390/polym12051171)
Supplement: Supplementary file 1 [file polymers-12-01171-s001.pdf]

SUPPLEMENTARY INFORMATION

Microstructural Contributions of Different Polyolefins to the Deformation Mechanisms of Their Binary Blends

Astrid Van Belle [1,†], Ruben Demets [1,2,†], Niclas Mys [1,2], Karen Van Kets [1], Jo Dewulf [3], Kevin Van Geem [4], Steven De Meester [2], and Kim Ragaert [1,*]

[1] Centre for Polymer and Material Technologies (CPMT), Department of Materials, Textiles and Chemical Engineering, Faculty of Engineering and Architecture, Ghent University, Technologiepark 130, B-9052 Zwijnaarde, Belgium; van.belle.astrid@gmail.com (A.V.B.); ruben.demets@ugent.be (R.D.); nicolas.mys@ugent.be (N.M.); karen.vankets@ugent.be (K.V.K.)

[2] Department of Green Chemistry and Technology, Faculty of Bioscience Engineering, Ghent University – Campus Kortrijk, Graaf Karel de Goedelaan 5, 8500 Kortrijk, Belgium; steven.demeester@ugent.be

[3] Sustainable Systems Engineering (STEN), Department of Green Chemistry and Technology, Faculty of Bioscience Engineering, Ghent University, Coupure Links 653, 9000 Ghent, Belgium; jo.dewulf@ugent.be

[4] Laboratory for Chemical Technology (LCT), Department of Materials, Textiles and Chemical Engineering, Faculty of Engineering and Architecture, Ghent University, Technologiepark 125, B-9052 Zwijnaarde, Belgium; kevin.vangeem@ugent.be

* Corresponding: kim.ragaert@ugent.be

† Both authors contributed equally to this manuscript.

INJECTION MOULDING PARAMETERS

LLDPE + LDPE									
Amount of LDPE (w%)	**100**	**95**	**90**	**80**	**50**	**20**	**10**	**5**	**0**
Temperatures (°C)									
1	160	160	160	160	160	160	160	160	160
2	170	170	170	170	170	170	170	170	170
3	180	180	180	180	180	180	180	180	180
4 (nozzle)	190	190	190	190	190	190	190	190	190
Deviation temperaturen (°C)	15	15	15	15	15	15	15	15	15
Temperature mould (°C)	15	15	15	15	15	15	15	15	15
Injection speed (mm/s)	A1	A1	A1	A1	D1	D1	D2	D2	D2
Holding pressure (bar)	400	400	400	450	550	600	500	500	500
Time holding pressure (s)	2	2	2	2	5	5	5	5	5
Cooling time (s)	20	20	20	20	20	20	20	20	20
Dosing length (mm)	70	70	70	70	70	70	70	70	70
Decompression after dosing (mm)	5	5	5	5	5	5	5	5	5
Dosing speed (%)	60	60	60	60	60	60	60	60	60
Dosing pressure (bar)	400	400	400	400	400	400	400	400	400
Switching point (mm)	12	12	12	12	10	8	10	10	10

HDPE + LDPE									
Amount of LDPE (w%)	**100**	**95**	**90**	**80**	**50**	**20**	**10**	**5**	**0**
Temperatures (°C)									
1	160	160	160	160	160	160	160	160	160
2	170	170	170	170	170	170	170	170	170
3	180	180	180	180	180	180	180	180	180
4 (nozzle)	190	190	190	190	190	190	190	190	190

Deviation temperaturen (°C)	15	15	15	15	15	15	15	15	15
Temperature mould (°C)	15	15	15	15	15	15	15	15	15
Injection speed (mm/s)	A1	A1	A1	A1	C1	C1	C1	C1	C1
Holding pressure (bar)	400	400	400	400	300	350	350	350	350
Time holding pressure (s)	2	2	2	2	2	2	2	2	2
Cooling time (s)	20	20	20	20	20	20	20	20	20
Dosing length (mm)	70	70	70	70	70	70	70	70	70
Decompression after dosing (mm)	5	5	5	5	5	5	5	5	5
Dosing speed (%)	60	60	60	60	60	60	60	60	60
Dosing pressure (bar)	400	400	400	400	400	400	400	400	400
Switching point (mm)	12	12	12	12	10	10	10	10	10

PP + LDPE									
Amount of LDPE (w%)	**100**	**95**	**90**	**80**	**50**	**20**	**10**	**5**	**0**
Temperatures (°C)									
1	200	200	200	200	200	200	200	200	200
2	210	210	210	210	210	210	210	210	210
3	220	220	220	220	220	220	220	220	220
4 (nozzle)	230	230	230	230	230	230	230	230	230
Deviation temperaturen (°C)	15	15	15	15	15	15	15	15	15
Temperature mould (°C)	15	10	10	10	10	10	10	10	10
Injection speed (mm/s)	A2	B1	B1	B1	B2	B2	B2	B3	B4
Holding pressure (bar)	400	450	475	500	550	550	575	575	600
Time holding pressure (s)	2	4	4	4	4	4	4	4	5
Cooling time (s)	20	30	30	30	30	30	30	30	30
Dosing length (mm)	70	70	70	70	70	70	70	70	70

Decompression after dosing (mm)	5	5	5	5	5	5	5	5	5
Dosing speed (%)	60	60	60	60	60	60	60	60	60
Dosing pressure (bar)	400	400	400	400	400	400	400	400	400
Switching point (mm)	12	14	14	14	14	14	14	14	13

LLDPE + PP									
Amount of PP (w%)	**100**	**95**	**90**	**80**	**50**	**20**	**10**	**5**	**0**
Temperatures (°C)									
1	200	200	200	200	200	200	200	200	200
2	210	210	210	210	210	210	210	210	210
3	220	220	220	220	220	220	220	220	220
4 (nozzle)	230	230	230	230	230	230	230	230	230
Deviation temperaturen (°C)	15	15	15	15	15	15	15	15	15
Temperature mould (°C)	10	15	15	15	15	15	15	15	15
Injection speed (mm/s)	B4	50	50	50	50	50	50	50	50
Holding pressure (bar)	600	500	500	500	500	500	900	900	900
Time holding pressure (s)	5	4	4	4	4	4	4	4	4
Cooling time (s)	30	20	20	20	20	20	20	20	20
Dosing length (mm)	70	70	70	70	70	70	70	70	70
Decompression after dosing (mm)	5	5	5	5	5	5	5	5	5
Dosing speed (%)	60	60	60	60	60	60	60	60	60
Dosing pressure (bar)	400	250	250	250	250	250	250	250	250
Switching point (mm)	13	12	12	12	12	12	12	12	12

HDPE + PP									
Amount of PP (w%)	**100**	**95**	**90**	**80**	**50**	**20**	**10**	**5**	**0**
Temperatures (°C)									

1	200	200	200	200	200	200	200	200	200
2	210	210	210	210	210	210	210	210	210
3	220	220	220	220	220	220	220	220	220
4 (nozzle)	230	230	230	230	230	230	230	230	230
Deviation temperaturen (°C)	15	15	15	15	15	15	15	15	15
Temperature mould (°C)	10	15	15	15	15	15	15	15	15
Injection speed (mm/s)	B4	50	50	50	30	20	20	20	20
Holding pressure (bar)	600	700	700	700	700	700	700	700	700
Time holding pressure (s)	5	4	4	4	4	4	4	4	4
Cooling time (s)	30	20	20	20	20	20	20	20	20
Dosing length (mm)	70	70	70	70	70	70	70	70	70
Decompression after dosing (mm)	5	5	5	5	5	5	5	5	5
Dosing speed (%)	60	60	60	60	60	60	60	60	60
Dosing pressure (bar)	400	250	250	250	250	250	250	250	250
Switching point (mm)	13	12	12	12	12	12	12	12	12

HDPE + LLDPE									
Amount of LLDPE (w%)	**100**	**95**	**90**	**80**	**50**	**20**	**10**	**5**	**0**
Temperatures (°C)									
1	160	160	160	160	160	160	160	160	160
2	170	170	170	170	170	170	170	170	170
3	180	180	180	180	180	180	180	180	180
4 (nozzle)	190	190	190	190	190	190	190	190	190
Deviation temperaturen (°C)	15	15	15	15	15	15	15	15	15
Temperature mould (°C)	15	15	15	15	15	15	15	15	15
Injection speed (mm/s)	D2	70	65	65	50	45	45	45	C1
Holding pressure (bar)	500	600	600	600	600	450	450	450	350

Time holding pressure (s)	5	5	5	5	5	5	5	5	2
Cooling time (s)	20	20	20	20	20	20	20	20	20
Dosing length (mm)	70	70	70	70	70	70	70	70	70
Decompression after dosing (mm)	5	5	5	5	5	5	5	5	5
Dosing speed (%)	60	60	60	60	60	60	60	60	60
Dosing pressure (bar)	400	250	250	250	250	250	250	250	400
Switching point (mm)	10	8	8	8	8	8	8	8	10

Injection speed settings:

Grafiek A1		Grafiek B4		Grafiek C1		Grafiek D1		Grafiek D2	
Position (mm)	Speed (mm/s)	Position (mm)	Speed (mm/s)	Position (mm)	Speed (mm/s)	Position (mm)	Speed (mm/s)	Position (mm)	Speed (mm/s)
0	70	0	70	0	30	0	90	0	90
28,7	70	28,7	70	8,82	60	10	110	10	110
47,79	60	47,79	60	26,47	70	30	130	30	130
75	55	75	55	35,29	80	40	150	40	150
				52,94	90	60	170	60	160
				75	110	75	200	75	170

MECHANICAL PROPERTIES OF THE INVESTIGATED BLENDS

Amount of LLDPE (wt%)	Amount of LDPE (wt%)	E (MPa)	σ_y (MPa)	ε_y (%)	ε_b (%)
0	100	192 ± 4	6.64 ± 0.09	3.08 ± 0.09	89.0 ± 2.0
5	95	171 ± 11	6.33 ± 0.22	3.29 ± 0.29	104.8 ± 5.4
10	90	168 ± 7	6.46 ± 0.14	3.39 ± 0.20	115.5 ± 4.2
20	80	159 ± 5	6.22 ± 0.11	3.42 ± 0.12	140.4 ± 7.5
50	50	139 ± 7	5.18 ± 0.14	3.16 ± 0.21	217.2 ± 11.4
80	20	128 ± 7	4.90 ± 0.10	3.19 ± 0.24	324.9 ± 44.8
90	10	129 ± 5	4.66 ± 0.10	2.98 ± 0.15	501.4 ± 15.9
95	5	126 ± 3	4.61 ± 0.09	3.00 ± 0.09	529.8 ± 21.0
100	0	114 ± 2	4.75 ± 0.05	3.41 ± 0.07	556.5 ± 11.6

Amount of HDPE (wt%)	Amount of PP (wt%)	E (MPa)	σ_y (MPa)	ε_y (%)	ε_b (%)
0	100	1771 ± 28	37.11 ± 0.17	8.09 ± 0.06	68.5 ± 9.5
5	95	1635 ± 35	36.55 ± 0.28	7.66 ± 0.07	222.8 ± 67.0
10	90	1592 ± 24	35.82 ± 0.30	7.66 ± 0.05	217.0 ± 74.2
20	80	1515 ± 27	34.02 ± 0.50	7.85 ± 0.14	106.1 ± 18.6
50	50	1280 ± 34	29.61 ± 0.82	6.80 ± 0.64	6.8 ± 0.7
80	20	1061 ± 32	25.86 ± 0.43	6.40 ± 0.45	6.4 ± 0.5
90	10	1034 ± 35	25.24 ± 0.24	7.52 ± 1.59	8.2 ± 1.7
95	5	994 ± 34	25.09 ± 0.13	7.69 ± 0.11	12.5 ± 3.3
100	0	864 ± 51	23.83 ± 0.38	10.62 ± 0.14	268.9 ± 56.8

Amount of LLDPE (wt%)	Amount of HDPE (wt%)	E (MPa)	σ_y (MPa)	ε_y (%)	ε_b (%)
0	100	953 ± 33	24.34 ± 0.15	9.77 ± 0.08	463.0 ± 109.8
5	95	884 ± 24	22.96 ± 0.33	10.36 ± 0.11	264.4 ± 60.3
10	90	796 ± 38	21.73 ± 0.44	10.76 ± 0.18	582.1 ± 101.0
20	80	687 ± 23	19.34 ± 0.25	11.62 ± 0.25	675.0 ± 27.5

50	50	433 ± 21	14.46 ± 0.23	14.70 ± 0.22	416.6 ± 26.1
80	20	209 ± 19	5.36 ± 0.53	2.26 ± 0.38	432.8 ± 48.0
90	10	176 ± 12	4.66 ± 0.26	2.22 ± 0.28	454.1 ± 45.5
95	5	132 ± 6	4.54 ± 0.14	2.83 ± 0.21	490.4 ± 17.0
100	0	114 ± 2	4.75 ± 0.05	3.41 ± 0.07	556.5 ± 11.6

Amount of LLDPE (wt%)	Amount of PP (wt%)	E (MPa)	σ_y (MPa)	ε_y (%)	ε_b (%)
0	100	1771 ± 28	37.11 ± 0.17	8.09 ± 0.06	68.5 ± 9.5
5	95	1652 ± 31	35.56 ± 0.30	7.98 ± 0.08	66.4 ± 16.5
10	90	1533 ± 38	33.72 ± 0.44	8.17 ± 0.13	96.3 ± 27.9
20	80	1358 ± 29	30.95 ± 0.36	8.98 ± 0.20	227.5 ± 27.0
50	50	790 ± 43	21.81 ± 0.77	13.55 ± 0.80	607.3 ± 100.0
80	20	286 ± 39	7.00 ± 0.59	2.26 ± 0.11	563.7 ± 34.5
90	10	153 ± 5	6.02 ± 0.07	3.07 ± 0.07	462.3 ± 19.6
95	5	127 ± 5	5.71 ± 0.08	3.39 ± 0.08	469.8 ± 16.5
100	0	101 ± 2	5.26 ± 0.03	3.79 ± 0.08	427.0 ± 23.3

Amount of LDPE (wt%)	Amount of HDPE (wt%)	E (MPa)	σ_y (MPa)	ε_y (%)	ε_b (%)
0	100	953 ± 33	24.34 ± 0.15	9.77 ± 0.08	463.0 ± 109.8
5	95	871 ± 29	23.36 ± 0.20	10.24 ± 0.13	216.8 ± 25.5
10	90	860 ± 22	22.52 ± 0.14	10.55 ± 0.07	223.0 ± 62.9
20	80	699 ± 35	20.16 ± 0.19	11.83 ± 0.18	264.9 ± 204.1
50	50	462 ± 35	16.45 ± 0.22	14.51 ± 0.28	101.9 ± 10.2
80	20	292 ± 37	7.47 ± 0.89	2.49 ± 0.63	103.2 ± 3.7
90	10	238 ± 19	6.54 ± 0.78	2.55 ± 0.56	100.1 ± 5.1
95	5	200 ± 19	6.39 ± 0.55	2.90 ± 0.51	93.2 ± 4.4
100	0	192 ± 4	6.64 ± 0.09	3.08 ± 0.09	89.0 ± 2.0

Amount of LDPE (wt%)	Amount of PP (wt%)	E (MPa)	σ_y (MPa)	ε_y (%)	ε_b (%)

0	100	1771 ± 28	37.11 ± 0.17	8.09 ± 0.06	68.5 ± 9.5
5	95	1761 ± 44	36.37 ± 0.35	7.52 ± 0.08	85.3 ± 31.1
10	90	1627 ± 32	34.18 ± 0.24	7.75 ± 0.05	116.5 ± 19.5
20	80	1459 ± 27	31.84 ± 0.25	7.72 ± 0.10	134.4 ± 19.1
50	50	927 ± 41	23.69 ± 0.37	10.52 ± 0.23	60.3 ± 8.4
80	20	447 ± 23	7.99 ± 0.26	1.75 ± 0.13	124.6 ± 12.5
90	10	274 ± 19	6.58 ± 0.28	2.22 ± 0.21	111.2 ± 6.6
95	5	208 ± 7	6.47 ± 0.10	2.78 ± 0.13	106.6 ± 3.6
100	0	172 ± 4	7.51 ± 0.10	3.57 ± 0.10	114.3 ± 3.2

DSC RESULTS OF THE INVESTIGATED BLENDS

The DSC results are presented in the table below. The crystallinity percentages were calculated using following equation:

$$X_c(\%) = \frac{\Delta H_m - \Delta H_{cc}}{\Delta H_m^{\infty} \cdot x} \cdot 100\%$$

In which ΔH_m and ΔH_{cc} are the enthalpy of melt and cold crystallization of the polymers and x the weight fraction of the polymer in the blend.

The prediction lines were constructed using the rule of mixtures (parallel model).

A	A (w%)	B	B (w%)	Prediction X_c A (%)	X_c A (%)	Prediction X_c B (%)	X_c B (%)	$X_{c,\ total}$ (%)
HDPE	0	PP	100	0.00	0.00	42.19	42.19	42.19
HDPE	5	PP	95	3.18	5.96	40.08	38.57	44.52
HDPE	10	PP	90	6.37	8.91	37.97	40.02	48.93
HDPE	20	PP	80	12.73	17.39	33.75	37.68	55.07
HDPE	50	PP	50	31.83	33.70	21.09	18.08	51.77
HDPE	80	PP	20	50.92	58.24	8.44	0.00	58.24
HDPE	90	PP	10	57.29	64.49	4.22	0.00	64.49
HDPE	95	PP	5	60.47	60.72	2.11	0.78	61.49
HDPE	100	PP	0	63.65	63.65	0.00	0.00	63.65
LDPE	0	HDPE	100	0.00	0.00	65.07	65.07	65.07
LDPE	5	HDPE	95	1.63		61.81		64.44
LDPE	10	HDPE	90	3.27		58.56		62.94
LDPE	20	HDPE	80	6.54		52.05		61.09

LDPE	50	HDPE	50	16.34	20.78	32.53	28.29	49.08
LDPE	80	HDPE	20	26.14	24.29	13.01	15.12	39.42
LDPE	90	HDPE	10	29.41	25.74	6.51	10.54	36.28
LDPE	95	HDPE	5	31.04	28.96	3.25	5.55	34.51
LDPE	100	HDPE	0	32.68	32.68	0.00	0.00	32.68
LDPE	0	LLDPE	100					28.79
LDPE	5	LLDPE	95					28.33
LDPE	10	LLDPE	90					28.81
LDPE	20	LLDPE	80					29.66
LDPE	50	LLDPE	50					30.26
LDPE	80	LLDPE	20					31.24
LDPE	90	LLDPE	10					32.20
LDPE	95	LLDPE	5					34.23
LDPE	100	LLDPE	0	No deconvolution of peaks possible				32.68
LDPE	0	PP	100	0.00	0.00	42.19	42.19	42.19
LDPE	5	PP	95	1.69	1.03	40.08	36.67	37.70
LDPE	10	PP	90	3.38	2.99	37.97	42.39	45.38
LDPE	20	PP	80	6.75	6.56	33.75	35.21	41.78
LDPE	50	PP	50	16.88	15.69	21.09	18.67	34.36
LDPE	80	PP	20	27.00	27.42	8.44	7.16	34.58
LDPE	90	PP	10	30.38	30.28	4.22	3.31	33.59
LDPE	95	PP	5	32.07	31.59	2.11	1.69	33.28
LDPE	100	PP	0	33.76	33.76	0.00	0.00	33.76
LLDPE	0	HDPE	100					65.07
LLDPE	5	HDPE	95	No deconvolution of the peaks possible				68.09
LLDPE	10	HDPE	90					56.06
LLDPE	20	HDPE	80					58.99

LLDPE	50	HDPE	50					46.08
LLDPE	80	HDPE	20					35.82
LLDPE	90	HDPE	10					35.38
LLDPE	95	HDPE	5					35.41
LLDPE	100	HDPE	0					28.79
LLDPE	0	PP	100	0.00	0.00	42.19	42.19	42.19
LLDPE	5	PP	95	1.45	0.00	40.08	37.88	37.88
LLDPE	10	PP	90	2.91	6.57	37.97	44.20	50.77
LLDPE	20	PP	80	5.82	8.14	33.75	39.03	47.17
LLDPE	50	PP	50	14.55	14.32	21.09	18.23	32.55
LLDPE	80	PP	20	23.28	24.76	8.44	5.48	30.24
LLDPE	90	PP	10	26.18	27.04	4.22	3.68	30.72
LLDPE	95	PP	5	27.64	29.55	2.11	1.22	30.77
LLDPE	100	PP	0	29.09	29.09	0.00	0.00	29.09

HDPE/PP 5 95

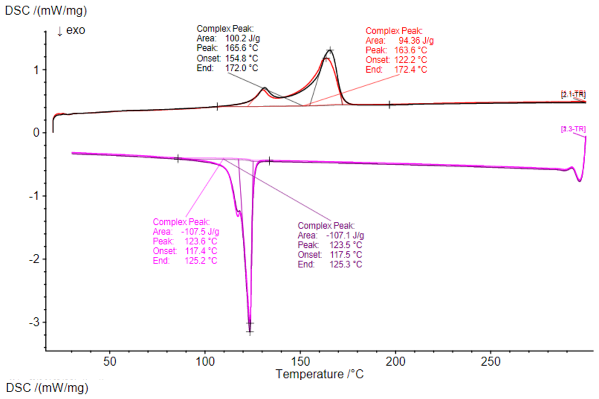

HDPE/PP 10 90

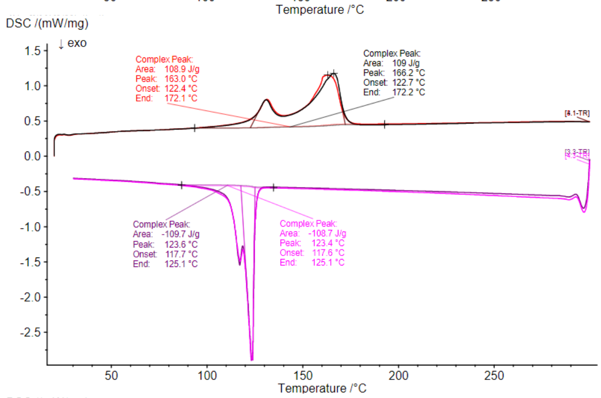

HDPE/PP 20 80

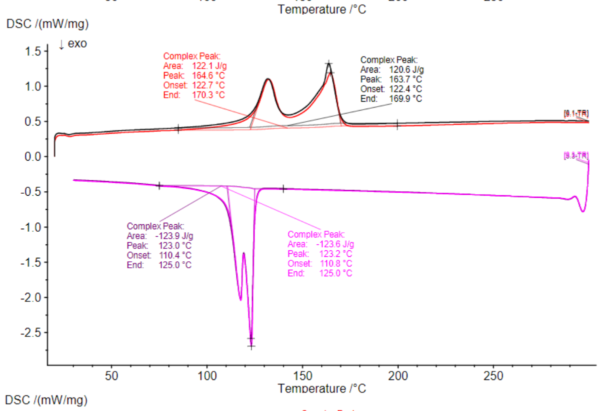

HDPE/PP 50 50

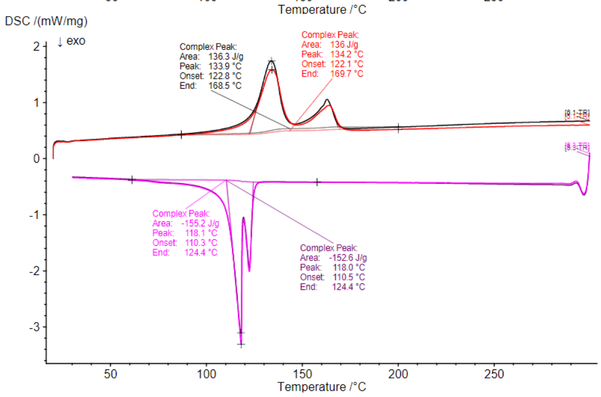

HDPE/PP	80	20	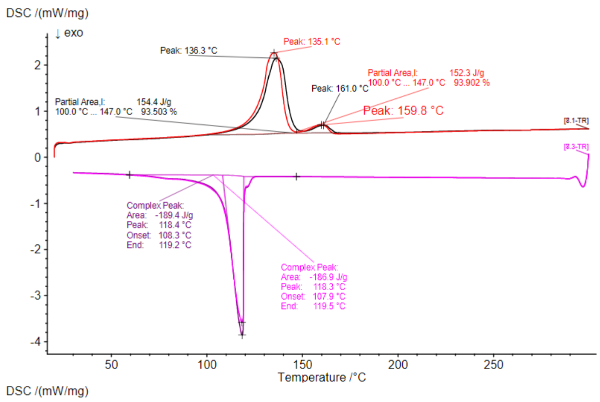
HDPE/PP	90	10	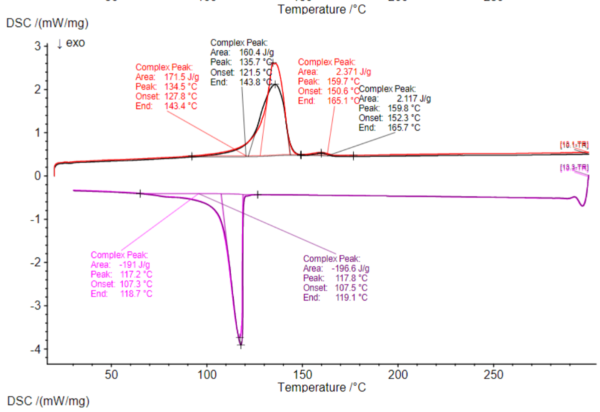
HDPE/PP	95	5	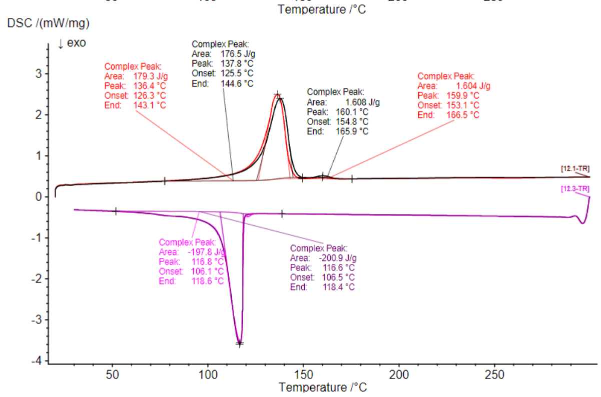
LDPE/HDPE	5	95	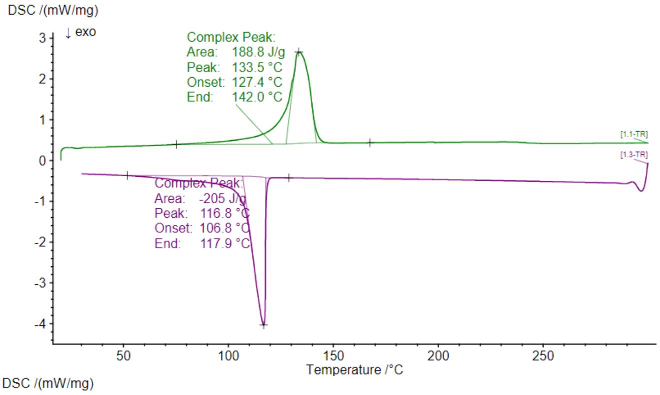
LDPE/HDPE	10	90	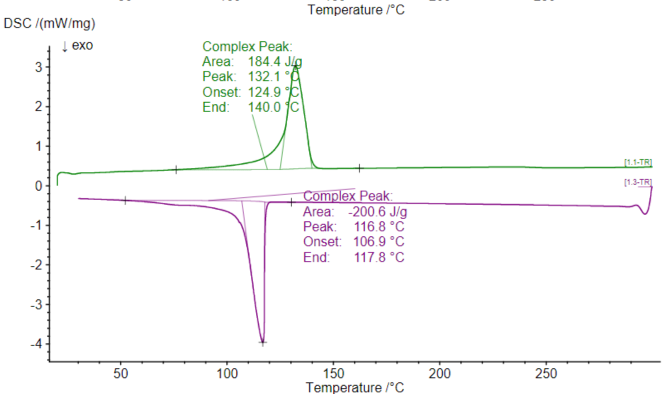

LDPE/HDPE	20	80	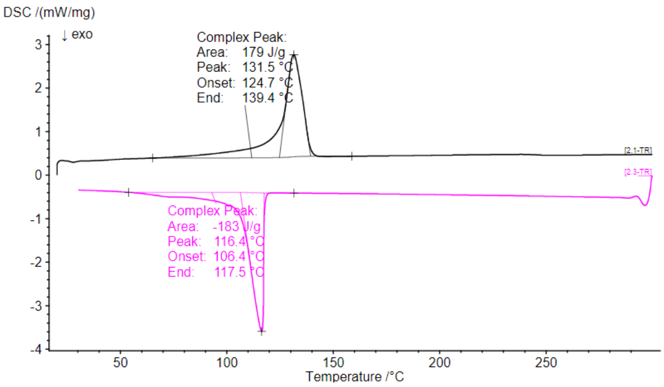
LDPE/HDPE	50	50	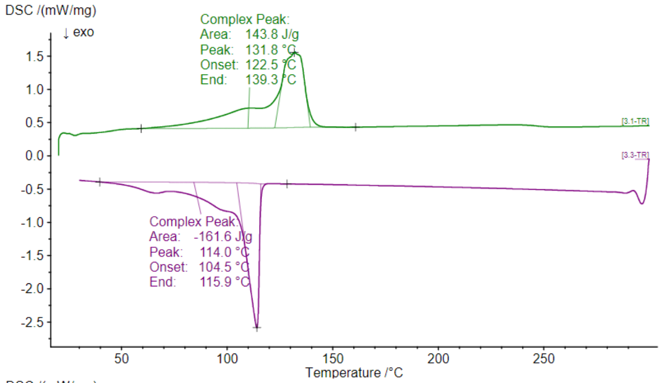
LDPE/HDPE	80	20	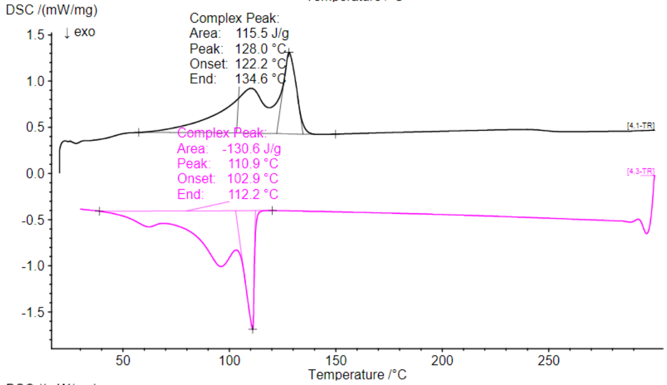
LDPE/HDPE	90	10	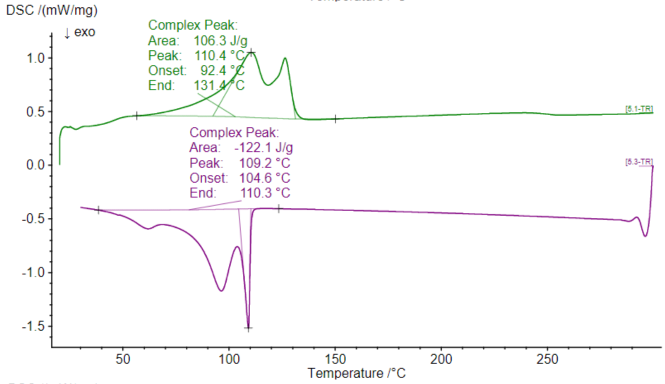
LDPE/HDPE	95	5	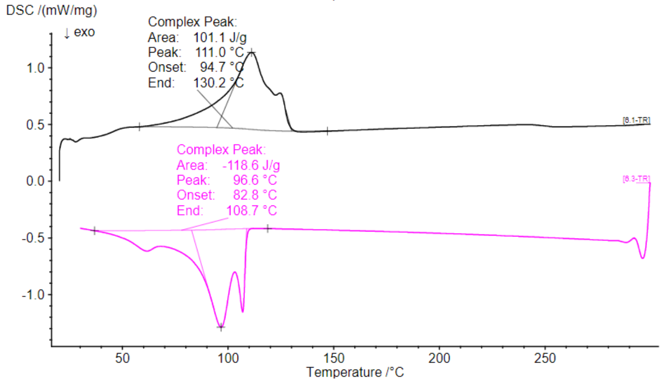

LDPE/LLDPE 5 95

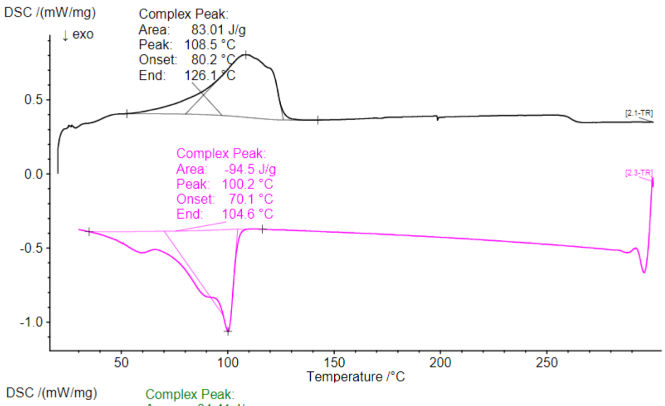

LDPE/LLDPE 10 90

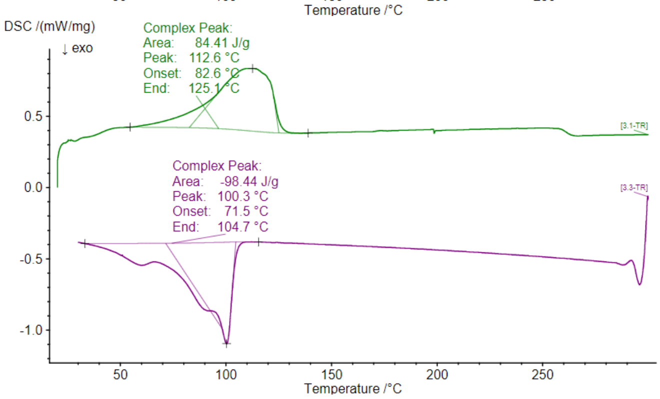

LDPE/LLDPE 20 80

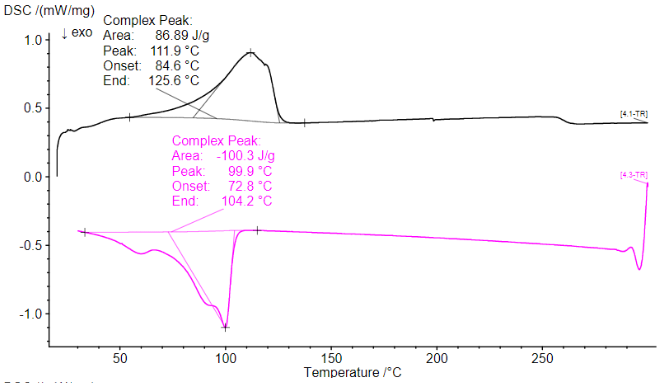

LDPE/LLDPE 50 50

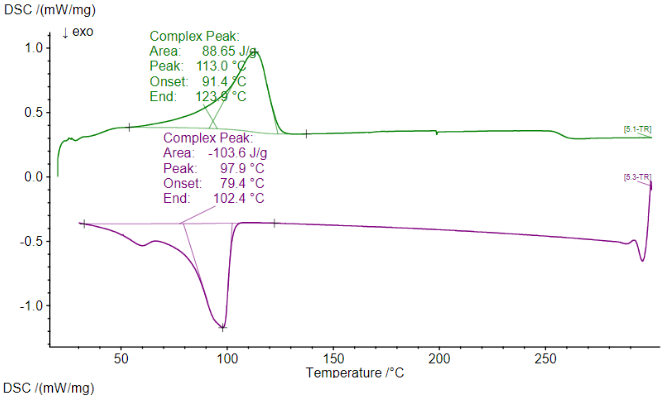

LDPE/LLDPE 80 20

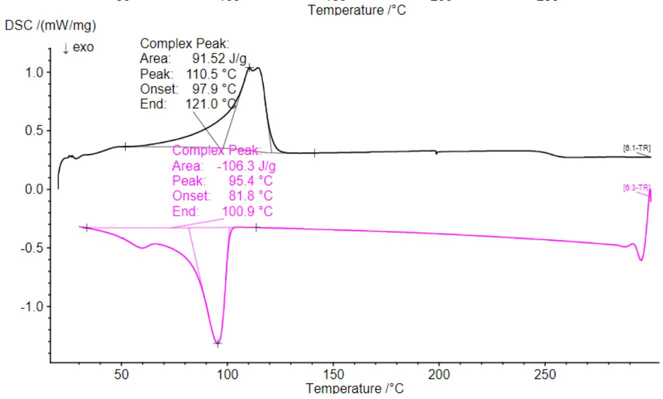

LDPE/LLDPE	90	10	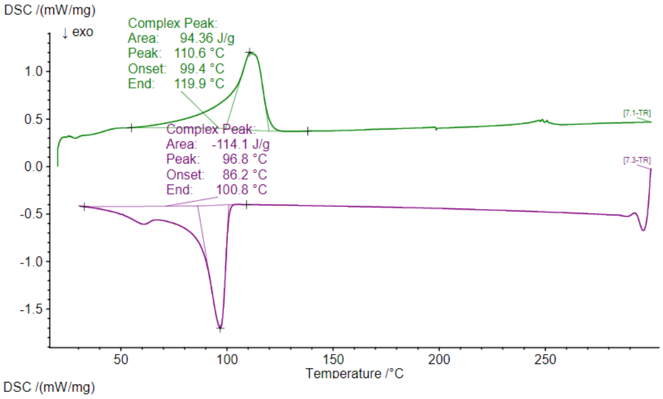
LDPE/LLDPE	95	5	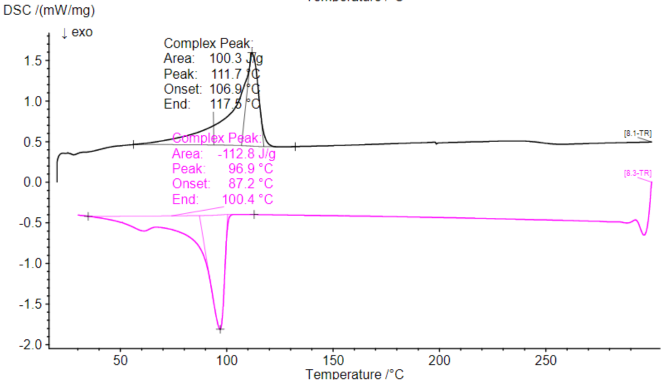
LDPE/PP	5	95	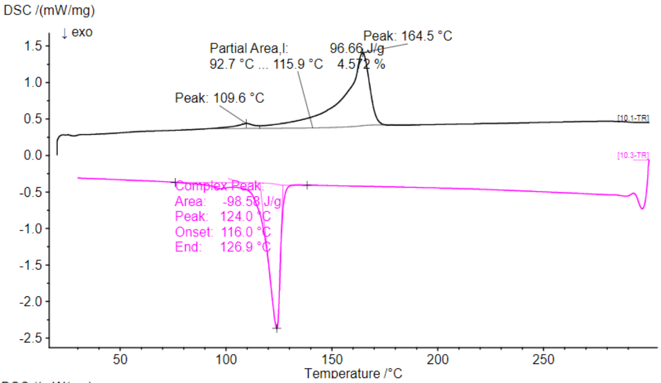
LDPE/PP	10	90	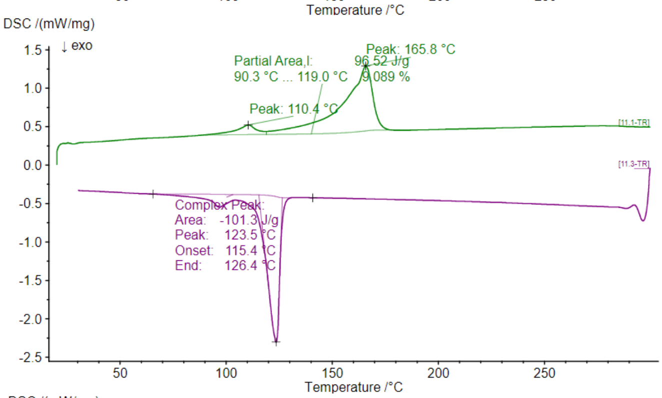
LDPE/PP	20	80	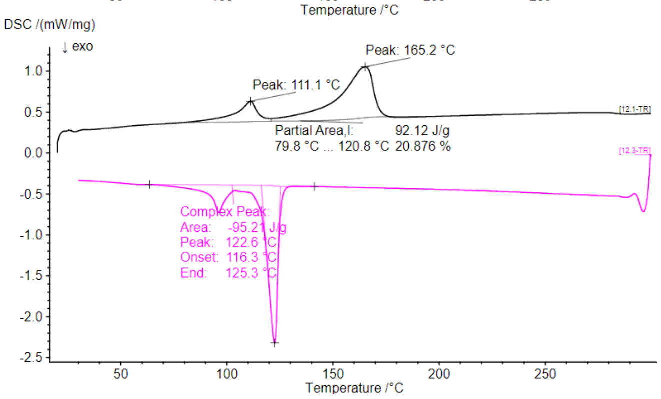

LDPE/PP	50	50

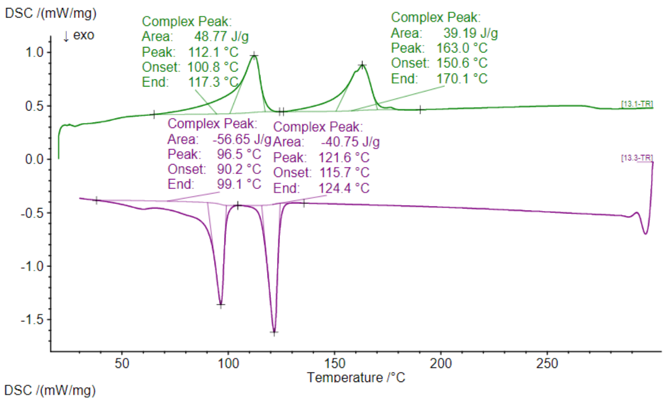

LDPE/PP	80	20

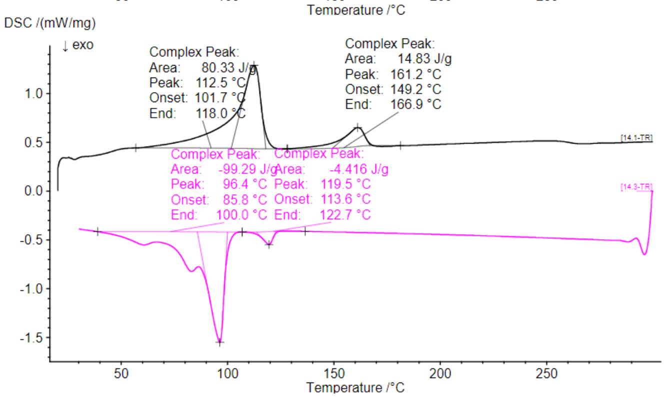

LDPE/PP	90	10

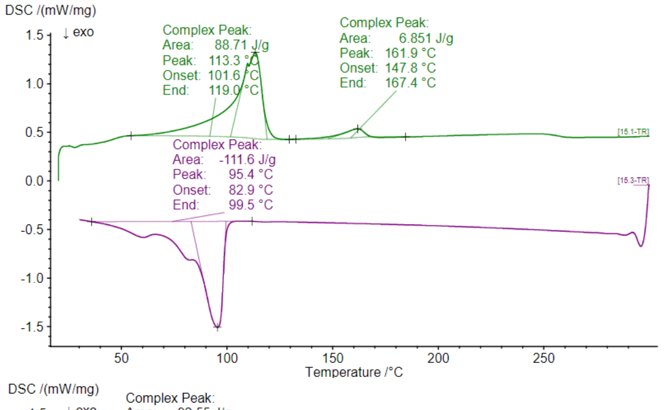

LDPE/PP	95	5

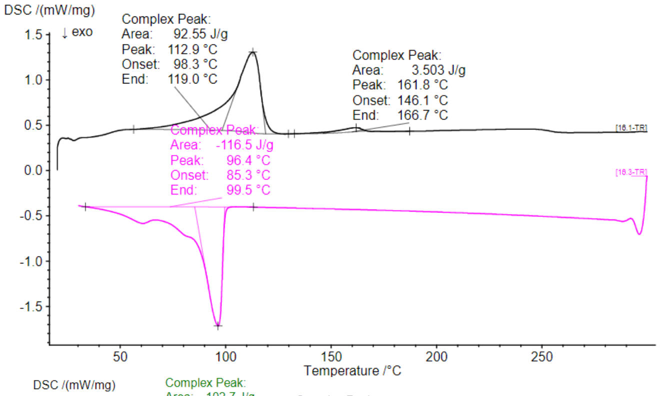

HDPE/LLDPE	5	95

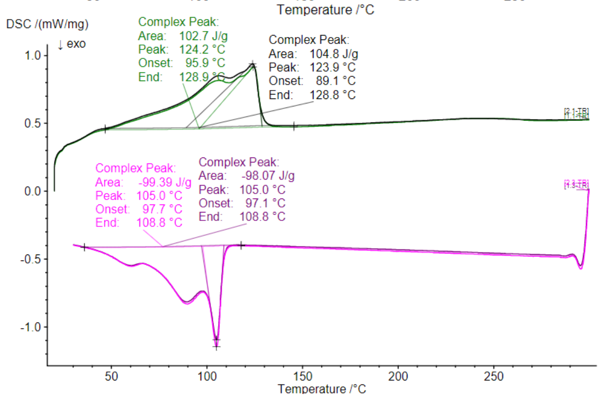

<table>
<tr><td>HDPE/LLDPE</td><td>10</td><td>90</td><td>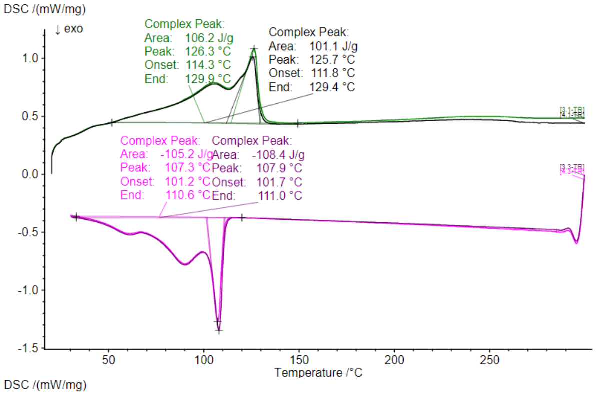
</td></tr>
<tr><td>HDPE/LLDPE</td><td>20</td><td>80</td><td>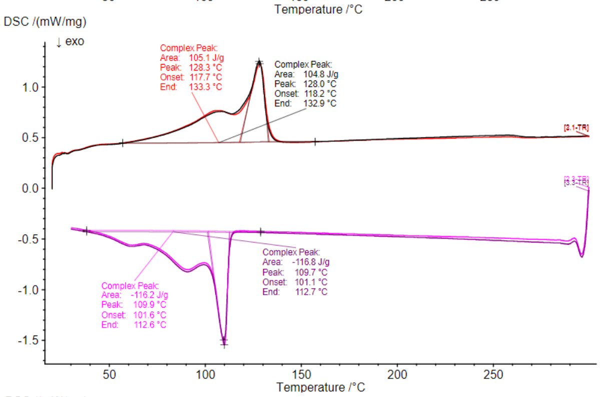
</td></tr>
<tr><td>HDPE/LLDPE</td><td>50</td><td>50</td><td>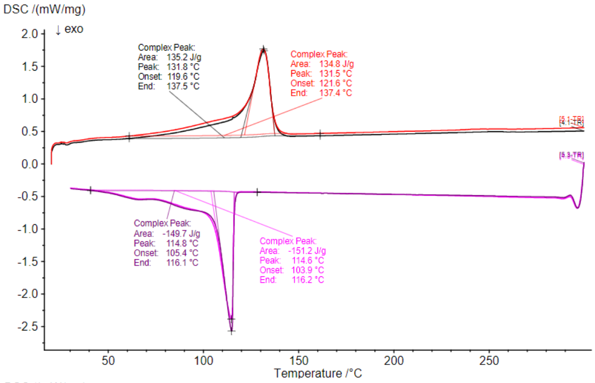
</td></tr>
<tr><td>HDPE/LLDPE</td><td>80</td><td>20</td><td>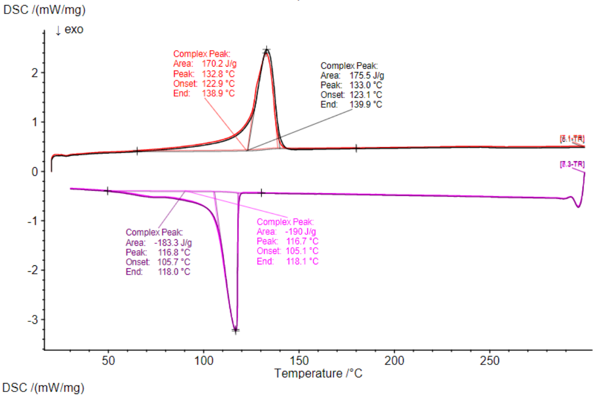
</td></tr>
<tr><td>HDPE/LLDPE</td><td>90</td><td>10</td><td>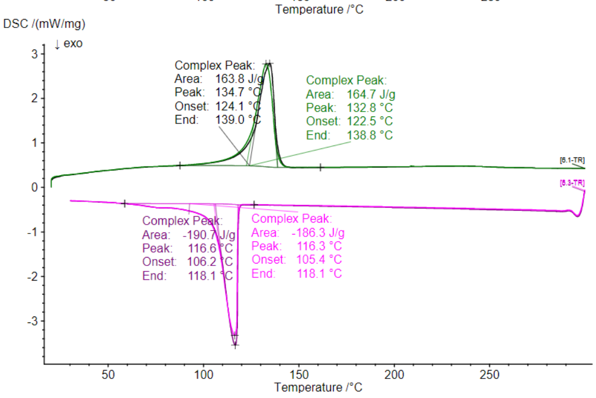
</td></tr>
</table>

<table>
<tr><td>HDPE/LLDPE</td><td>95</td><td>5</td><td>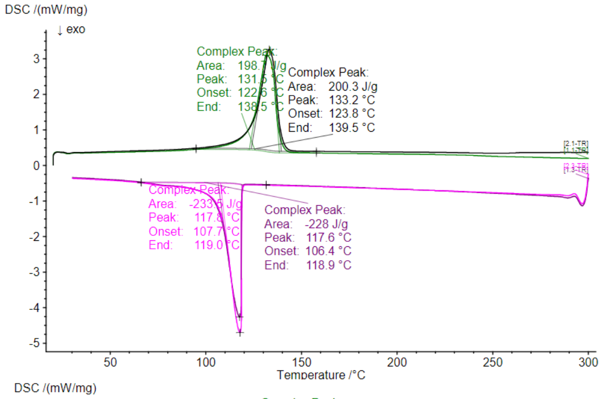
</td></tr>
<tr><td>LLDPE/PP</td><td>5</td><td>95</td><td>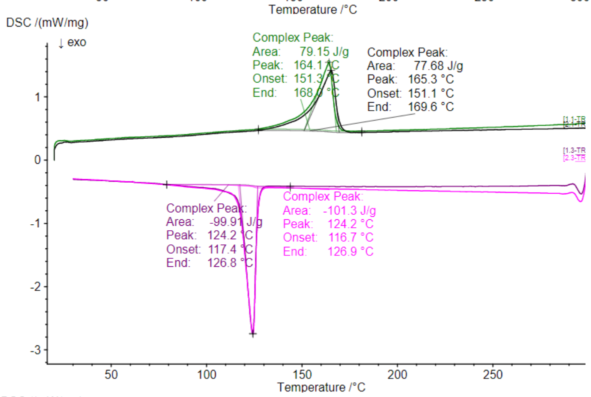
</td></tr>
<tr><td>LLDPE/PP</td><td>10</td><td>90</td><td>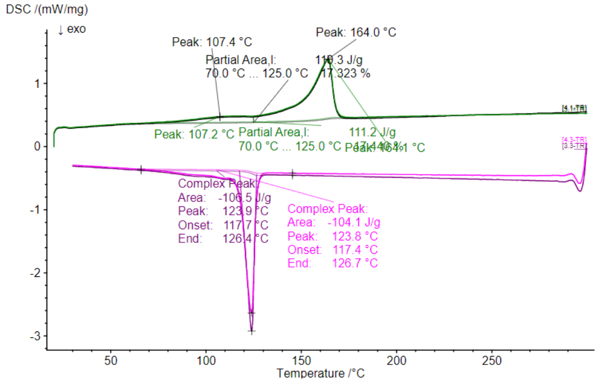
</td></tr>
<tr><td>LLDPE/PP</td><td>20</td><td>80</td><td>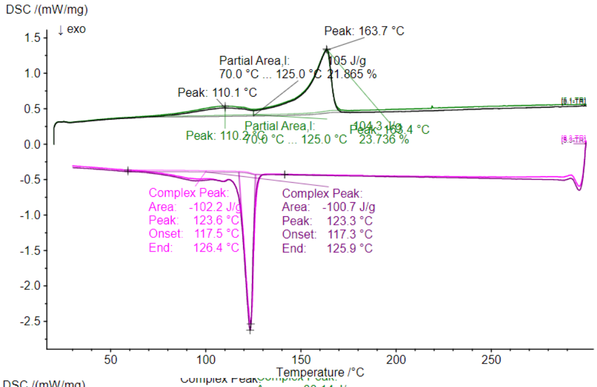
</td></tr>
<tr><td>LLDPE/PP</td><td>50</td><td>50</td><td>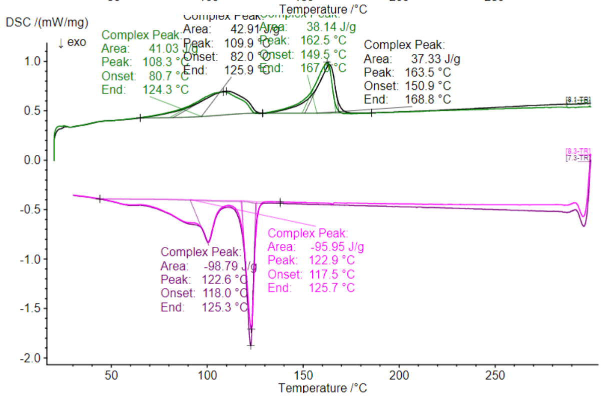
</td></tr>
</table>

LLDPE/PP 80 20

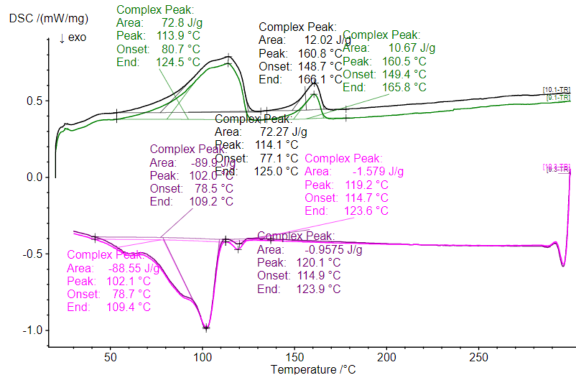

LLDPE/PP 90 10

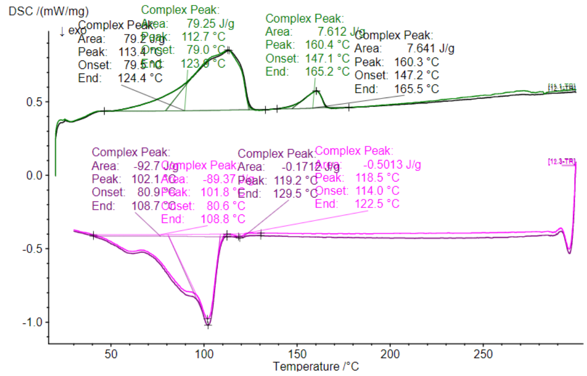

LLDPE/PP 95 5

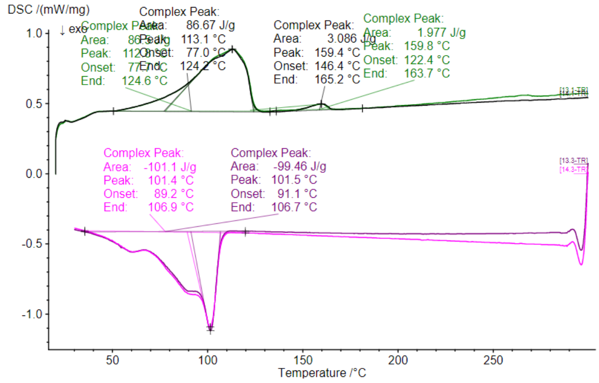

PICTURES OF THE TESTED SAMPLES

The pictures below show the macroscopic fibrillation observed during the execution of the tensile tests of the PP and HDPE polymers. The microscope used is a Keyence VXH500.

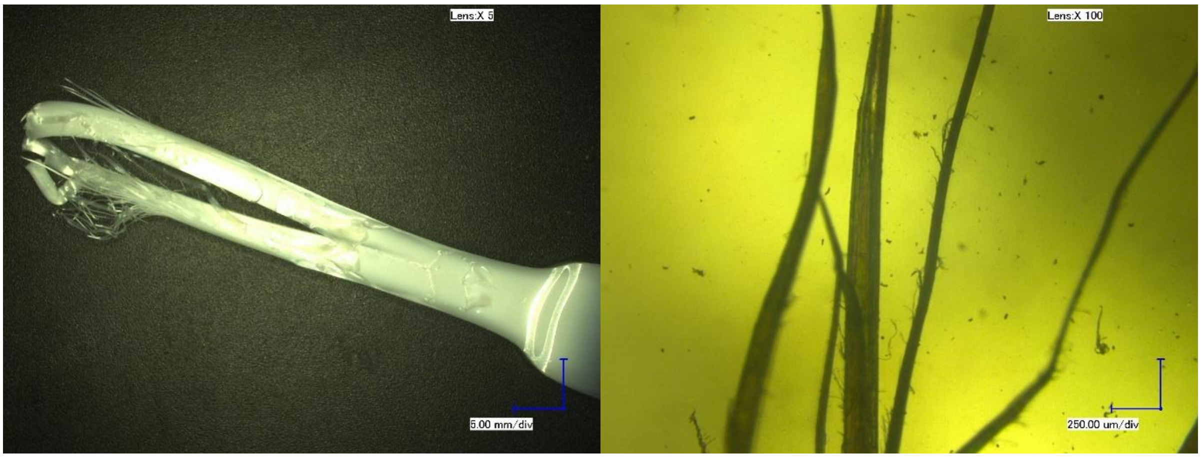

Figure 1: Macroscopic fibrillitation of PP: (left picture) Magnification x5; (right picture) Magnification x100.

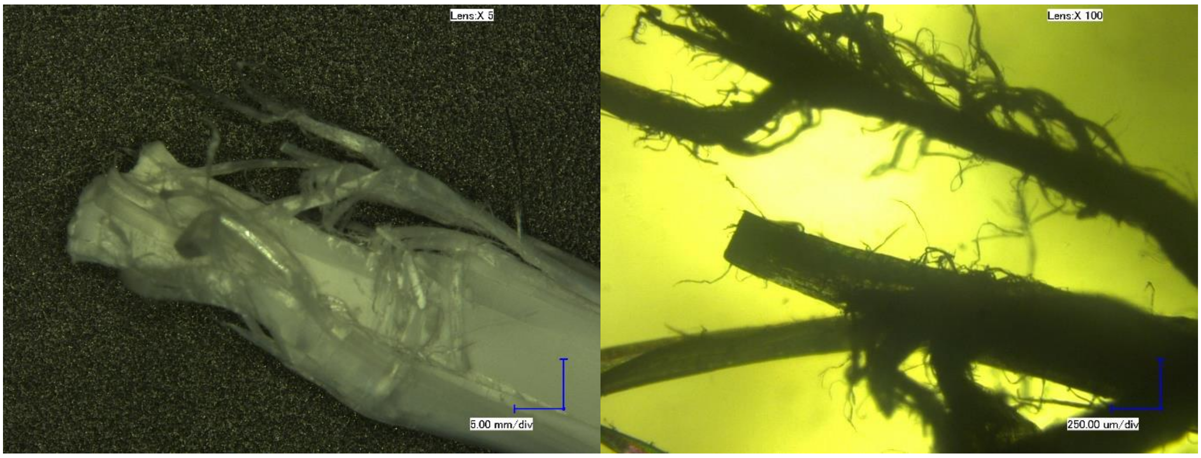

Figure 2: Macroscopic fibrillation of HDPE: (left picture) Magnification x5; (right picture) Magnification x100.

Following Table shows the visual representations of the test bars next to the photos of the real tested samples:

Code	Curve	Dog bone deformation	Photos of tested samples	
A	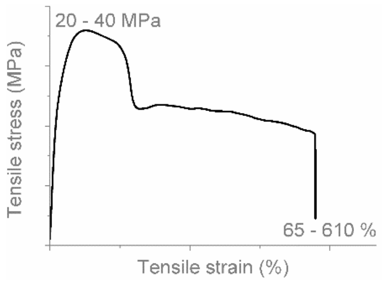	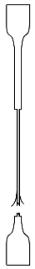	HDPE	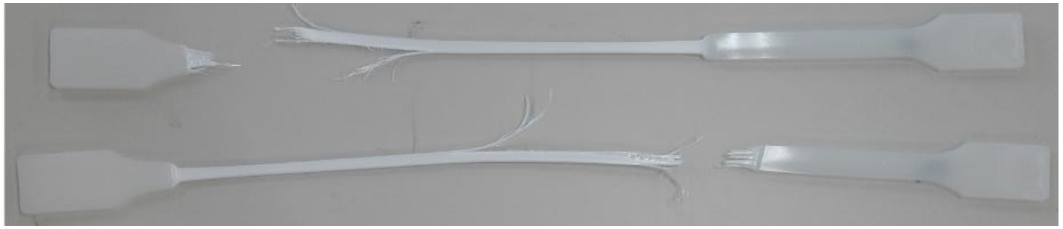
			PP	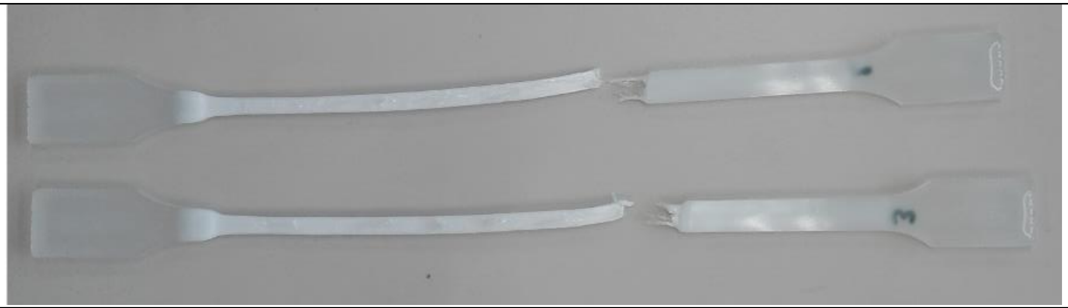
B	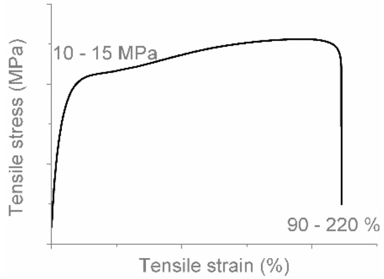	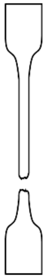	LDPE	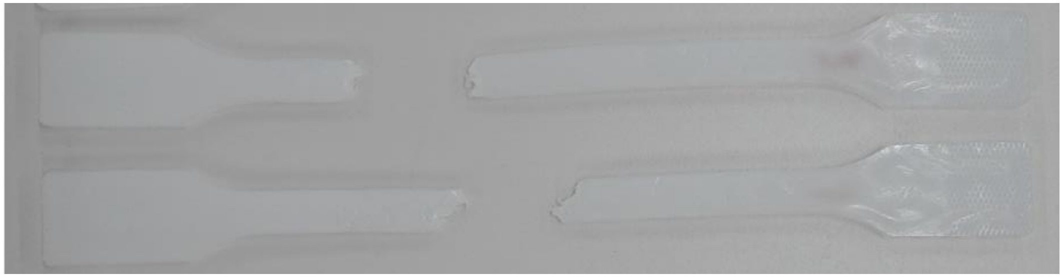

C	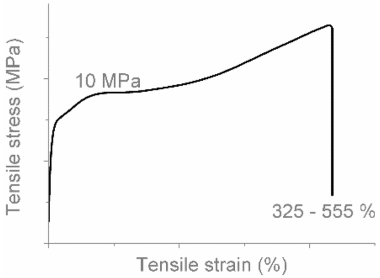	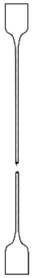	LLDPE	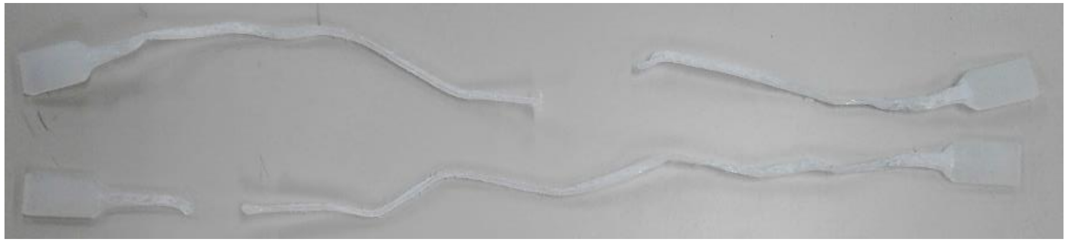
AB	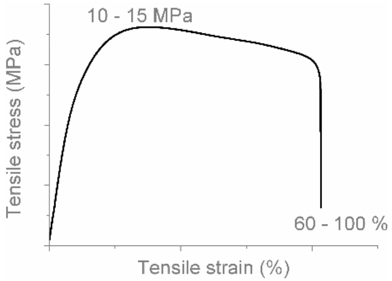	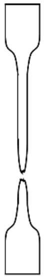	LDPE<br>+<br>HDPE<br><br>50/50	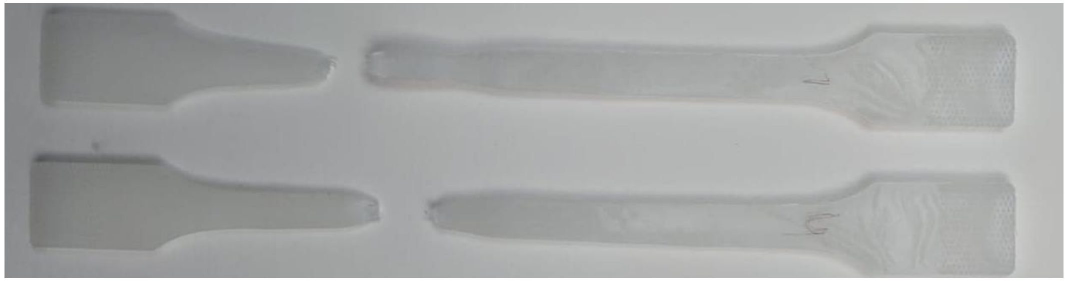
AC	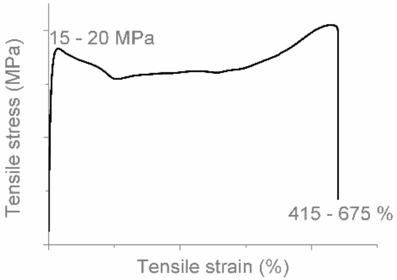	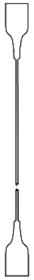	HDPE<br>+<br>LLDPE<br><br>50/50	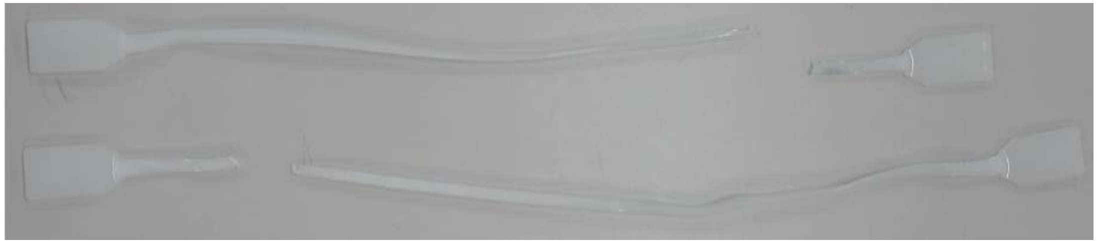

AA	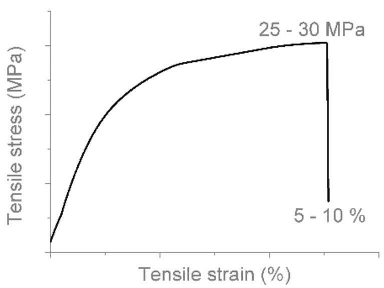	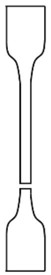	HDPE + PP 80/20	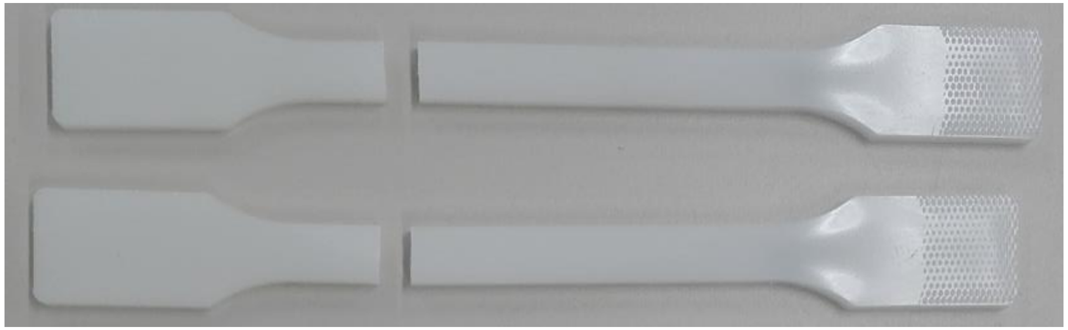
